# Multimorbidity analysis with low condition counts: a robust Bayesian approach for small but important subgroups

**DOI:** 10.1016/j.ebiom.2024.105081

**Published:** 2024-03-21

**Authors:** Guillermo Romero Moreno, Valerio Restocchi, Jacques D. Fleuriot, Atul Anand, Stewart W. Mercer, Bruce Guthrie

**Affiliations:** a School of Informatics, University of Edinburgh, Edinburgh, UK; b Centre for Cardiovascular Science, University of Edinburgh, Edinburgh, UK; c Usher Institute of Population Health Sciences and Informatics, University of Edinburgh, Edinburgh, UK

**Keywords:** Multimorbidity, Association measures, Network analysis, Relative risk, Bayesian inference, Low counts

## Abstract

**Background:**

Robustly examining associations between long-term conditions may be important in identifying opportunities for intervention in multimorbidity but is challenging when evidence is limited. We have developed a Bayesian inference framework that is robust to sparse data and used it to quantify morbidity associations in the oldest old, a population with limited available data.

**Methods:**

We conducted a retrospective cross-sectional study of a representative dataset of primary care patients in Scotland as of March 2007. We included 40 long-term conditions and studied their associations in 12,009 individuals aged 90 and older, stratified by sex (3039 men, 8970 women). We analysed associations obtained with Relative Risk (RR), a standard measure in the literature, and compared them with our proposed measure, Associations Beyond Chance (ABC). To enable a broad exploration of interactions between long-term conditions, we built networks of association and assessed differences in their analysis when associations are estimated by RR or ABC.

**Findings:**

Our Bayesian framework was appropriately more cautious in attributing association when evidence is lacking, particularly in uncommon conditions. This caution in reporting association was also present in reporting differences in associations between sex and affected the aggregated measures of multimorbidity and network representations.

**Interpretation:**

Incorporating uncertainty into multimorbidity research is crucial to avoid misleading findings when evidence is limited, a problem that particularly affects small but important subgroups. Our proposed framework improves the reliability of estimations of associations and, more in general, of research into disease mechanisms and multimorbidity.

**Funding:**

10.13039/501100000272National Institute for Health and Care Research.

**Research in context**

**Evidence before this study**

We conducted two searches on PubMed (May 4, 2023). The first search focused on the network analysis of multimorbidity, using the terms ‘network’ AND ‘multimorbidity’ OR ‘comorbidity’, including wild card definitions. Existing studies use varied measures of association and methodologies to determine if associations are statistically significant. A weakness is that many do not report confidence intervals for associations or otherwise appropriately account for uncertainty, for example when comparing sub-populations or studying properties of the multimorbidity networks. Potential distortions caused by rare conditions are typically dealt with by excluding rare conditions from the study, which are defined using *ad hoc* thresholds of minimum prevalence or a minimum number of cases with no statistically based justification. No article used a statistically robust framework for the estimation of association. Moreover, most studies do not consider the impact of the combination of inaccuracies and low condition counts in their analysis. Finally, we found that most articles only compute pairwise (or at best ternary) associations, rather than examining all possible associations.

The second search concerned the use of Bayesian approaches for the analysis of multimorbidity, using the terms ‘Bayesian’ AND ‘multimorbidity’ OR ‘comorbidity’, including wild card definitions. The listed works generally employed Bayesian causality networks. Although this approach is statistically robust and properly accounts for uncertainty, it requires assumptions that may not be realistic in the context of multimorbidity. For instance, it enforces the presence of directionality in the associations (while these may not be clear from the data) and does not allow loops, neglecting the possibilities of different pathways through the causal networks.

**Added value of this study**

We have highlighted the issue of low condition counts in the estimation of associations and have provided methods to address it in a statistically robust manner that reduces the risk of reporting inaccurate or non-significant results (false positives). We have developed a Bayesian framework that provides explicit measures of uncertainty in the estimation of associations and that can be used for determining their significance or performing statistically robust comparisons among subgroups. Furthermore, the explicit measures of uncertainty can also be transferred to more sophisticated measures of multimorbidity, such as combined multimorbidity scores for single long-term conditions. Additionally, our model considers the effect of all other associations in the network when estimating any pairwise association, discounting for complex associations between multiple long-term conditions. Lastly, assumptions are explicit in our framework, so that they can be scrutinised and easily modified to facilitate the development of extensions and future improvements.

**Implications of all the available evidence**

Robustly examining associations between health conditions is crucial to advance the understanding of multimorbidity, for example, to identify long-term conditions with shared aetiology, or to forecast future health needs as populations age. We developed a framework that improves the reliability of the estimation of multimorbidity associations when condition counts are low, which is particularly relevant for discovering and quantifying associations between infrequent conditions and in small but important subgroups, for whom limited data availability can exacerbate health inequalities.

## Introduction

The prevalence of individuals living with two or more long-term conditions (multimorbidity) is increasing worldwide due to population ageing, improved survival from acute conditions, and changing patterns of risk factors like obesity. Over half of the English population over 65 years old were multimorbid in 2015 and this is projected to increase to two thirds by 2035.^1^ Many healthcare systems have developed around the management of single-organ conditions, with clinical pathways that are poorly suited to increasingly complex patients, and fewer resources are devoted to understanding the interaction of multiple long-term conditions. Our understanding of multimorbidity could be improved by uncovering underlying mechanisms behind co-occurring health conditions. While age is typically accepted as a determinant of multimorbidity, the reality behind the co-occurrence of conditions is complex. Some conditions increase the risk of other conditions, whereas others share common risk factors, such as genetic predisposition, patient behaviour, or environmental factors.^2^ Electronic Health Records (EHR) provide a rich source of population data that can be used to systematically understand multimorbidity from an epidemiological approach.^2^^,^^3^ In these datasets, robust measures of association between conditions are hypothesis-generating of potential causal relations if their co-occurrence is higher than expected.^4^

However, pairwise associations alone are insufficient for understanding multimorbidity, which is characterised by complex interactions between multiple health conditions. To facilitate a comprehensive exploration of these interactions, associations between conditions can be assembled into a network that depicts a unified landscape of multimorbidity.^5^, ^6^, ^7^ Network analysis can then be used to understand the role of specific diseases,^8^ find clusters of related conditions^8^, ^9^, ^10^ or study disease progression.^11^, ^12^, ^13^

However, the prevalent methodological frameworks employed in multimorbidity studies are often based on assumptions that falter when condition counts are low.^4^ Such scenarios render findings unreliable, risking the inflation of low numbers and reporting false positives.^7^ This is further amplified in the case of relative risk, a widely adopted measure, due to known biases that amplify associations among low-prevalence conditions.^6^^,^^10^^,^^14^ Analogous issues persist with other common measures of association such as Pearson's correlations or odds ratios.^4^^,^^15^^,^^16^ These problems may be further intensified when associations are aggregated to perform broader analyses of multimorbidity, such as in network analyses or ‘multimorbidity coefficients’.^17^

Importantly, the problems with low condition counts are not equally distributed in the population, as its impact is bigger on underrepresented populations for whom fewer patients are available in the datasets. These cohorts are usually analysed when a comparison of sub-populations is sought via stratification. The aim of this study was therefore to address these issues by developing and implementing a Bayesian framework that naturally incorporates uncertainty and facilitates the assessment of the statistical significance of disease associations for both common and rare conditions, and important (but often small) subgroups. Associations between conditions are highly likely to vary with age, so age-stratified analyses are necessary but are potentially problematic in small studies or for population sub-groups with small numbers even in large studies. This is the case of the oldest old, which is a small subpopulation group despite their high rates of multimorbidity, so we apply our Bayesian framework to robustly analyse their multimorbidity profile and compare results with those obtained by relative risk.

## Methods

### Study design and population

We conducted a cross-sectional analysis of a primary care dataset for 12,009 patients aged 90 years and over, alive and permanently registered at any of the participating 314 medical practices in Scotland as of March 2007. These data are a subset of a larger representative sample of about one-third of the Scottish population (1,751,841 patients).^18^ We used a list of 40 physical and mental long-term conditions (LTCs) as defined by a previous study of multimorbidity using this dataset (Supplementary Table S1).^19^ These LTCs ---which include both physical and mental conditions--- were selected for being identified as ‘core’ in multimorbidity measures or as important long-term disorders by NHS Scotland.^19^^,^^20^ We stratified this cohort by sex.

### Robust inference of associations

We developed a statistical Bayesian framework to examine associations between LTCs. This framework is based on a model of multimorbidity generation from i) hypothetical risk factors affecting the appearance of each LTC independent of the others *f*_*i*_, and ii) hypothetical common mechanisms that simultaneously affect a pair of LTCs and lead to their co-appearance *f*_*ij*_, e.g. common risk factors or one LTC increasing the risk of acquiring the other (Supplementary Section S1.2). This model can be linked to that of Rzhetsky et al.,^21^ where diseases appear due to the hypothetical accrual of deleterious polymorphisms in nucleotide sites that affect each disease independently or in nucleotide sites that affect both diseases simultaneously. However, our model is agnostic in the kind of shared mechanisms that result in association.

Our proposed association measure based on this generative framework, *Association Beyond Chance* (ABC), is conceptually similar to Relative Risk (RR). In the context of multimorbidity research, Relative Risk is often defined as the ratio between the number of times a pair of LTCs occurs and the product of their prevalence (which corresponds to the observed-to-expected ratio^14^), i.e. *RR*_*ij*_ = *N N*_*ij*_*/N*_*i*_*N*_*j*_, where *N* is the total number of patients in the cohort, *N*_*ij*_ is the count of patients having both conditions simultaneously, and *N*_*i*_ and *N*_*j*_ are the marginal counts of patients having each condition. Analogously, ABC is the ratio between co-appearance in association with common mechanisms and co-appearance by chance when related to *independent risk factors*, *ABC*_*ij*_ *= 1 + f*_*ij*_*/f*_*i*_ *f*_*j*_, where we have added one to the ratio to facilitate direct comparison to relative risk (Supplementary Section S1.2). As in RR, an ABC value equal to one reflects that there are no common mechanisms linking them and the two LTCs only co-appear by chance due to their independent factors, so they can be considered to be independent. Values of ABC above one suggest the presence of common mechanisms that increase the co-occurrence of both LTCs. For instance, an ABC of 2 reflects that the pair of LTCs appear together in association with common mechanisms as much as by chance from independent risk factors, or in other words the LTCs co-appear twice as much as expected by chance. On the contrary, values of ABC below one suggest the presence of mechanisms that hinder the co-occurrence of the LTCs, i.e. *negative associations*.

The values of ABC and independent factors are inferred from the data via Hamiltonian Monte Carlo sampling implemented with the statistical package *Stan* (Supplementary Section S1.3). Instead of providing single value estimates per association, the Bayesian framework returns samples of the posterior distributions, consequently providing an explicit quantification of uncertainty over the estimates. Unlike RR, ABC also incorporates the interaction with all other conditions in its computation, as we are constraining the inference of independent factors f_i_ to be shared across all interactions an LTC has. Therefore, the association between each pair of LTCs is not treated ‘in isolation’, which would correspond to a ‘purely pairwise’ or unconstrained model, having a separate pair of inferred independent factors *f*_*i*_ and *f*_*j*_ per pairwise association (as, for example, in the model by Rzhetsky et al. or in RR).^21^ Instead, our model infers a single *f*_*i*_ parameter that is shared across all associations that affect an LTC and better captures factors that are independent of any association with other conditions.

For each of our experiments, we drew 20,000 samples of the estimations and reported the middle value of the highest posterior density interval (i.e., the mode of these distributions, Supplementary Section S1.4) along with 99% equal-tailed credible intervals, corresponding to the 0.5 th and 99.5 th percentiles of the samples and we defined an association as not significant if its 99% credible interval includes the no-association outcome (ABC = 1). For RR associations, we used 99% confidence intervals using the Katz method and Fisher's exact test for assessing their significance (Supplementary Section S1.5). For evaluating significant differences in associations between sub-populations, we estimated the overlap between their distributions (Supplementary Section S1.6).

Our Bayesian approach also includes a prior belief that regulates the behaviour of extreme cases with just a few observations. Associations for which there are limited data are more susceptible to being influenced by the prior beliefs, while the influence of the prior belief vanishes as more empirical evidence is available. We selected the prior distribution for ABC to be centred around one, to bias the measure against the reporting of false positives. In this way, the ABC measure can be seen as simultaneously merging the value of the association with its significance, as association values will be shrunk if there is a lack of statistical evidence.^22^ We selected weakly informative priors regulated by hyperpriors for the remaining parameters (Supplementary Section S1.3).

### Analysis of multimorbidity

We integrated and synthesised the information about multimorbidity using two approaches. First, to enable a visual exploration of the complex interactions between multiple LTCs, we built network visualisations where nodes correspond to LTCs and edges reflect whether an association between two LTCs is significant, with edge width proportional to the association value. In these representations, clusters of related LTCs can be found with common community detection algorithms that optimally group conditions that have more connections between themselves than to other LTCs, which we find via the Clauset-Newman-Moore greedy modularity maximisation algorithm with resolution parameter equal to one.^23^ We then evaluate the quality of the found clusters via three measures: their *modularity* (which compares within-cluster links to what would be expected by chance), their *coverage* (which is the percentage of within-cluster edges), and their *performance* (which is the sum of within-cluster and potential but not existing between-cluster non-edges divided by the total number of possible edges). A higher number in each of these measures reflects a higher definition in the cluster partition.

Second, to explore the general tendency of individual LTCs to be associated with others, we report the average association of each LTC with all others. For ABC, average associations are computed for each sample of the posterior, resulting in a distribution of values. For RR, pairs with non-significant associations contribute to the average with an association value of 1, analogous to independence and, for simplicity, we report single-point estimates in the main text. Details of the computation of these measures can be found in Supplementary Section S1.7.

### Ethics

The NHS National Research Ethics Service had previously approved the anonymous use of these data for research purposes, therefore this study did not need individual ethics approval.

### Role of funding source

The funders of the study had no role in study design, data collection, data analysis, data interpretation, or writing of the report.

## Results

The oldest-old cohort (90 years old and above) in our population comprised 12,009 older people of whom 3039 (25.3%) were men and 8970 (74.7%) were women and whose age distribution can be found in Supplementary Figure S2. Of these individuals, 783 (6.5%) had no LTC diagnosed, 1444 (12.0%) had one LTC, 1968 (16.4%) had two LTCs, 2043 (17.0%) had three LTCs, and the remaining 5771 (48.1%) had four or more diagnosed LTCs (Supplementary Figure S3). The mean number of LTCs was 3.65 (SD 2.32) in the whole cohort, with significant differences between sexes (mean 3.43 [SD 2.35] in men and mean 3.72 [SD 2.30] in women, p < 0.001). The prevalence of the included LTCs varied in the cohort (Supplementary Table S1), from *Hypertension* (present in 5475 [45.6%]) to *Viral hepatitis*, which was present in <5 (<0.04%). The largest differences in prevalence between the sexes (excluding *Prostate disorders*, which only occur in men) were for *Hypertension* (37.6% of men vs 48.3% of women) and *Thyroid disorders* (10.1% of men vs 17.4% of women).

**Fig. 1:**
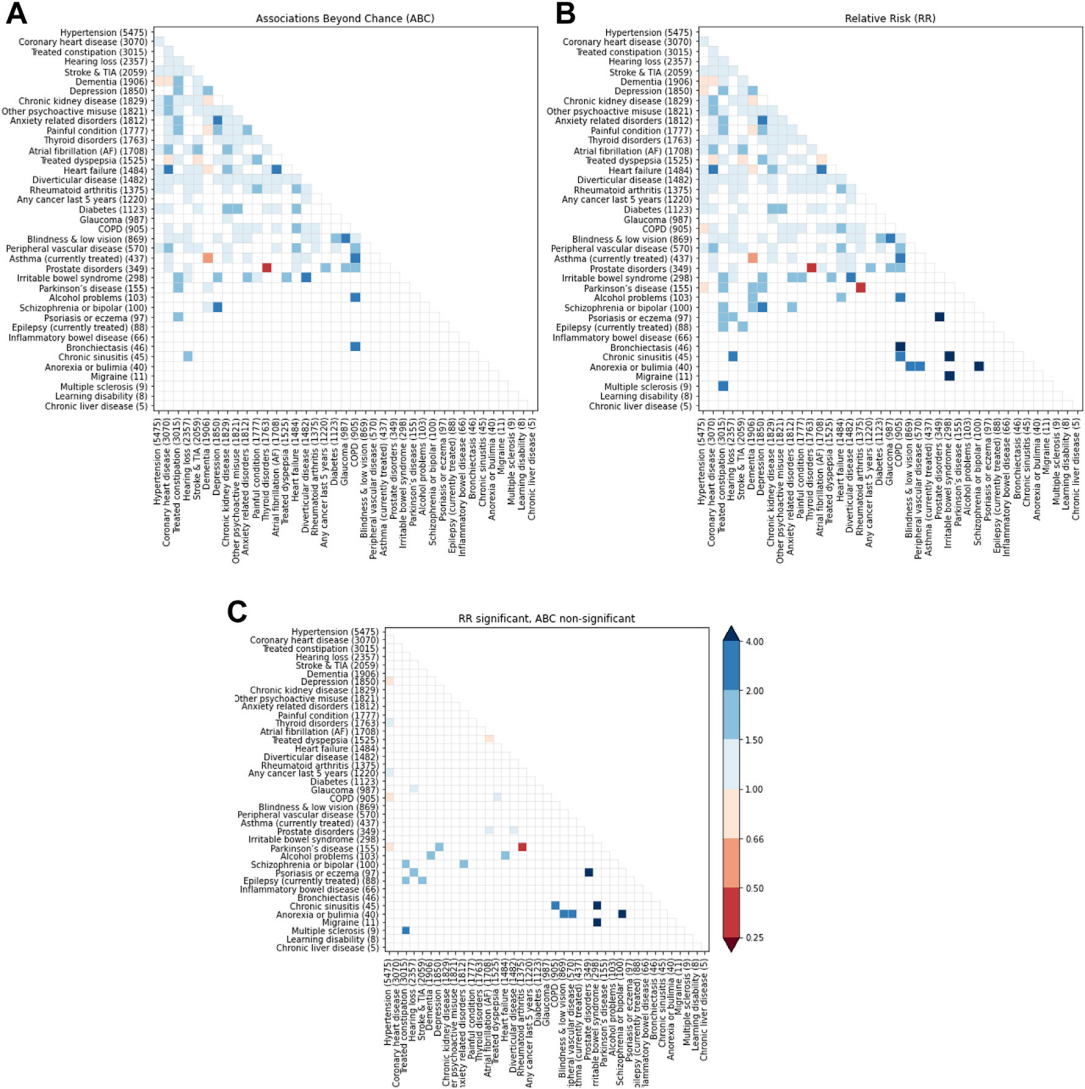
Associations found by ABC (A), RR (B), and RR but not ABC (C) between the studied 40 LTCs. Blue cells have significant positive associations (ABC or RR >1), red cells have significant negative associations (ABC or RR <1), and white cells have non-significant associations. Conditions are sorted by their prevalence in the cohort (with counts in parentheses), and association values are coloured by strength ranges, as indicated in the colour bar.

**Fig. 2:**
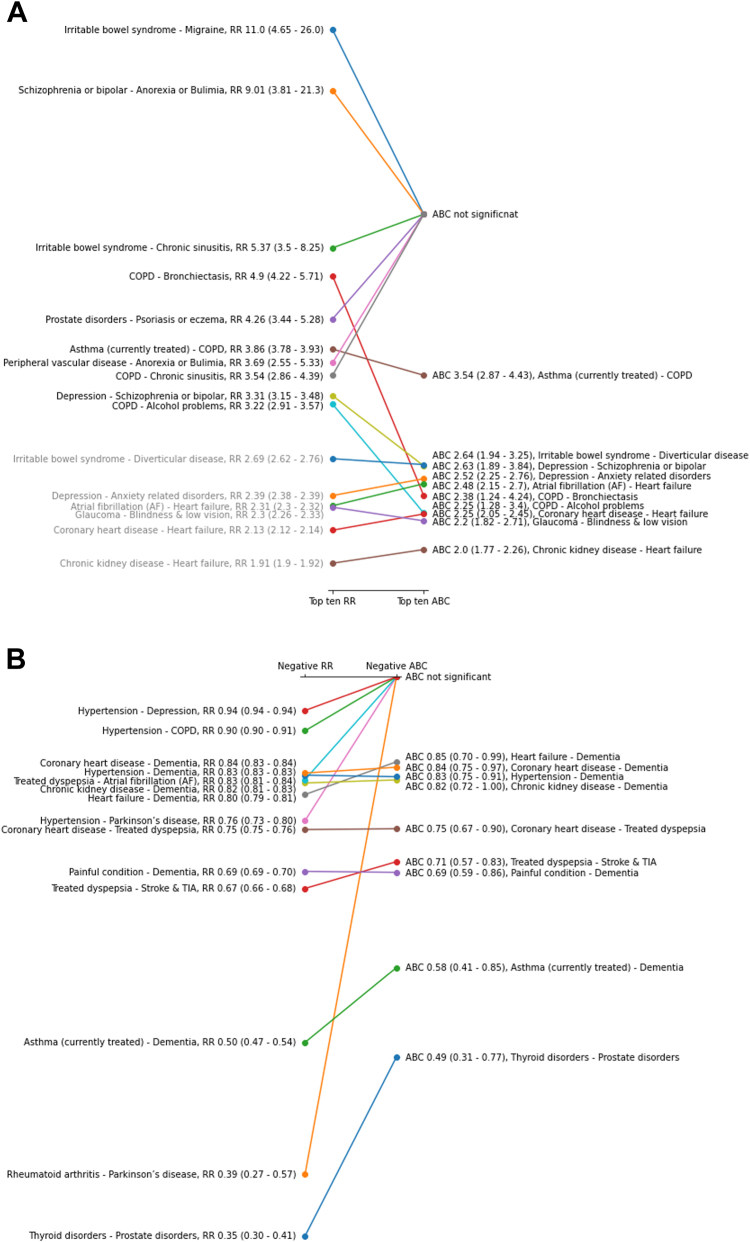
Top ten positive (A) and negative (B) associations found by RR (left) and ABC (right) in the oldest old. In grey, are associations that are not within the top ten of the measure but are within the top ten of the other measure. Parentheses give the 99% confidence intervals. Points follow a logarithmic scale.

We first analysed significant associations in the whole oldest-old cohort. Out of the 780 possible pairwise associations between the 40 LTCs, we found 192 (24.6%) and 167 (21.4%) significant associations using RR and ABC, respectively (Fig. 1A and B, Supplementary Table S2). Among these, 165 associations were found by both methods, 67 associations were found by RR only (28.9% of all found by RR) and two associations were only found by ABC (0.01% of all found by ABC). These discrepancies mostly occur for associations related to infrequent LTCs (Fig. 1C, Supplementary Figure S4). The top ten associations by RR and ABC can be seen in Fig. 2A. The highest positive associations found with RR occur between *Irritable bowel syndrome* and *Migraine,* and between *Schizophrenia, other psychosis or bipolar* and *Anorexia or bulimia*. The RR values of these associations (11.0 [99% CI 4.65–26.0) and 9.01 [99% CI 3.81–21.3]) are much higher than the median RR association value (1.34 [IQR 1.21–1.60]). Only four out of the top ten LTCs found by RR were also found to be significant by ABC, none of which belonged to the top three by RR. Conversely, all top ten associations found by ABC were found significant by RR, with the strongest associations corresponding to *Active asthma* with *COPD* (3.54 [2.87–4.43]) and *Irritable bowel syndrome* with *Diverticular disease* (2.64 [1.94–3.25]). Similar patterns can be found when looking at results stratified by sex, where ABC found significance in only three (in men) and two (in women) of the top ten associations found by RR (Supplementary Tables S7 and S8). The strongest association found by RR in men was between *Peripheral vascular disease* and *Anorexia or bulimia* (6.85 [99% CI 2.9–16.2]), and in women between *Alcohol problems* and *Learning disabilities* (55.4 [99% CI 15.2–202]), neither of which was found to be significant by ABC (Supplementary Figure S10).

Only 14 significant associations found by RR were negative (i.e. conditions occurred less often together than expected by chance [Fig. 2B]). Nine of these negative associations were also found by ABC, with no additional negative association found by ABC. Six of the negative associations found by both methods involved *Dementia*, and the strongest negative associations were between *Thyroid disorders* and *Prostate disorders* (both methods) and between *Rheumatoid and other connective tissue disorders* and *Parkinson's disease* (RR only, not significant by ABC). In men, only three negative associations were found by RR and one by ABC, whereas in women RR and ABC found twelve and five negative associations, respectively (Supplementary Table S10). As in the whole cohort, all negative associations found by ABC in men and women separately were also found by RR in these subgroups. The negative association between *Coronary heart disease* and *Treated dyspepsia* is the only one found by both methods in both men and women.

**Fig. 3:**
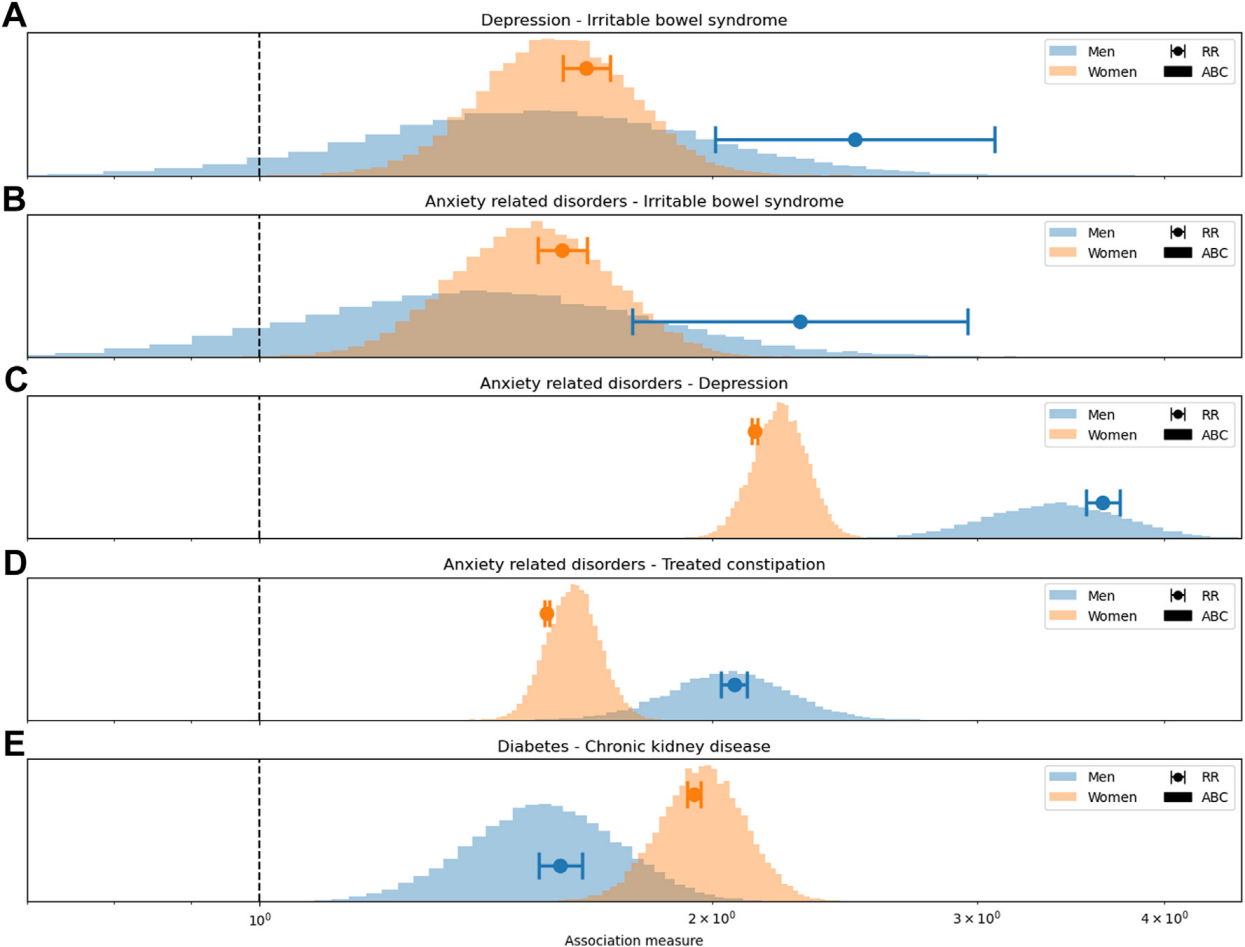
(A–E) Comparison of differences between associations by sex using ABC (shaded distributions) and RR (dots with error bars). Panels A and B show a separation between the RR central estimate and 99% confidence intervals between men (blue) and women (orange), while the overlapping distributions of association values obtained with ABC suggest that there is no significant difference between men and women for the two associations in those panels. Comparisons of the 79 associations found in both men and women by RR can be found in Supplementary Figures S5–S9.

When focusing on differences in associations between sexes, there also are disparities in results obtained with the two methods. There were 79 associations found significant in both men and women by RR and 56 by ABC. RR found that 47 (59.5%) out of the 79 associations were of different strength in men and women (Supplementary Tables S3 and S4), with large differences between *Depression* and *Irritable bowel syndrome*, and between *Anxiety-related disorders* and *Irritable bowel syndrome*, where RR was found to be higher in men (p-values <0.01, Fig. 3A and B). In contrast, ABC only found ten (17.9%) of the 56 associations to be of different strength in men and women, all of which are among the 47 also found by RR. For instance, ABC found that the associations between *Anxiety-related disorders* and *Depression* and between *Anxiety-related disorders* and *Treated constipation* were higher in men than in women (p < 0.01, Fig. 3C and D). Additionally, ABC found women to have a stronger association between *Diabetes* and *Chronic kidney disease* (p = 0.024, Fig. 3E). Note that ABC explicitly accounts for uncertainty while RR typically relies on confidence intervals under the assumption that the probability of appearance of a condition follows a normal distribution, which may not be appropriate when analysing less frequent LTCs, such as *Irritable bowel syndrome* (Fig. 3A and B).

**Fig. 4:**
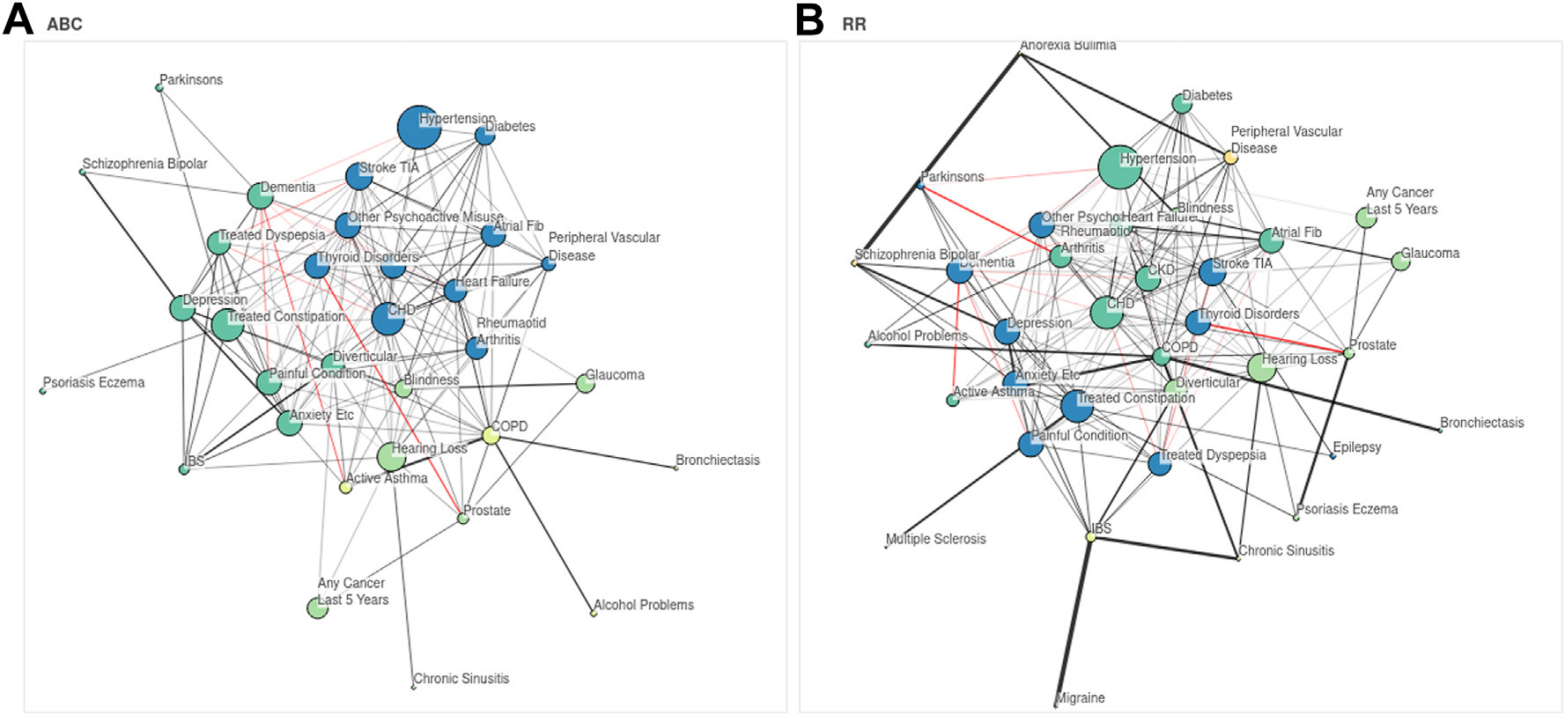
Networks of associations with ABC (A) and RR (B). Edge widths are proportional to the strength of the association (in logarithmic scale), with positive associations in black and negative associations in red. Nodes are coloured by their cluster allocation, as found by the community detection algorithm. The size of nodes is proportional to the square root of the prevalence of the LTCs. The network layout was built using the Kamada-Kawai path-length cost function.

We integrated the information about multimorbidity associations by RR and ABC into network representations, in which clusters of conditions can be found (Fig. 4). Community detection on the network of ABC associations (Fig. 4A) suggests the presence of two major clusters of LTCs, one comprising mental health conditions (*Depression*, *Painful condition*, *Anxiety related disorders, Dementia, Schizophrenia, other psychosis or bipolar, etc*) and another including cardiovascular and metabolic conditions (*Coronary heart disease*, *Heart failure*, *Atrial fibrillation*, *Peripheral vascular disease*, *Hypertension,* etc), and two smaller clusters, one mostly including respiratory conditions (*Active asthma*, *COPD*, *Bronchiectasis*, *Alcohol problems*) and another one including *Chronic sinusitis*, *Prostate*, *Glaucoma*, *Blindness*, *Hearing loss*, and *Cancer*. Community detection on the network from RR associations (Fig. 4B) suggests the presence of five clusters instead, with the cluster of respiratory conditions the cluster of circulatory and metabolic conditions merged, and two new clusters emerging, one with *Chronic sinusitis*, *Irritable bowel syndrome*, and *Migraine*, and another one with *Anorexia or bulimia*, *Schizophrenia, other psychosis or bipolar* and *Peripheral vascular disease*. These clusters present a weaker structure, as shown by the measures of cluster quality, which correspond to values of modularity of 0.18, coverage of 0.54, and performance of 0.76 for the ABC network and values of modularity of 0.14, coverage of 0.44, and performance of 0.73 for the RR network. A full list of the conditions in each cluster can be found in Supplementary Table S5. Similar disparities in clustering quality can be found on network visualisations after stratifying by sex (Supplementary Figure S11).

**Fig. 5:**
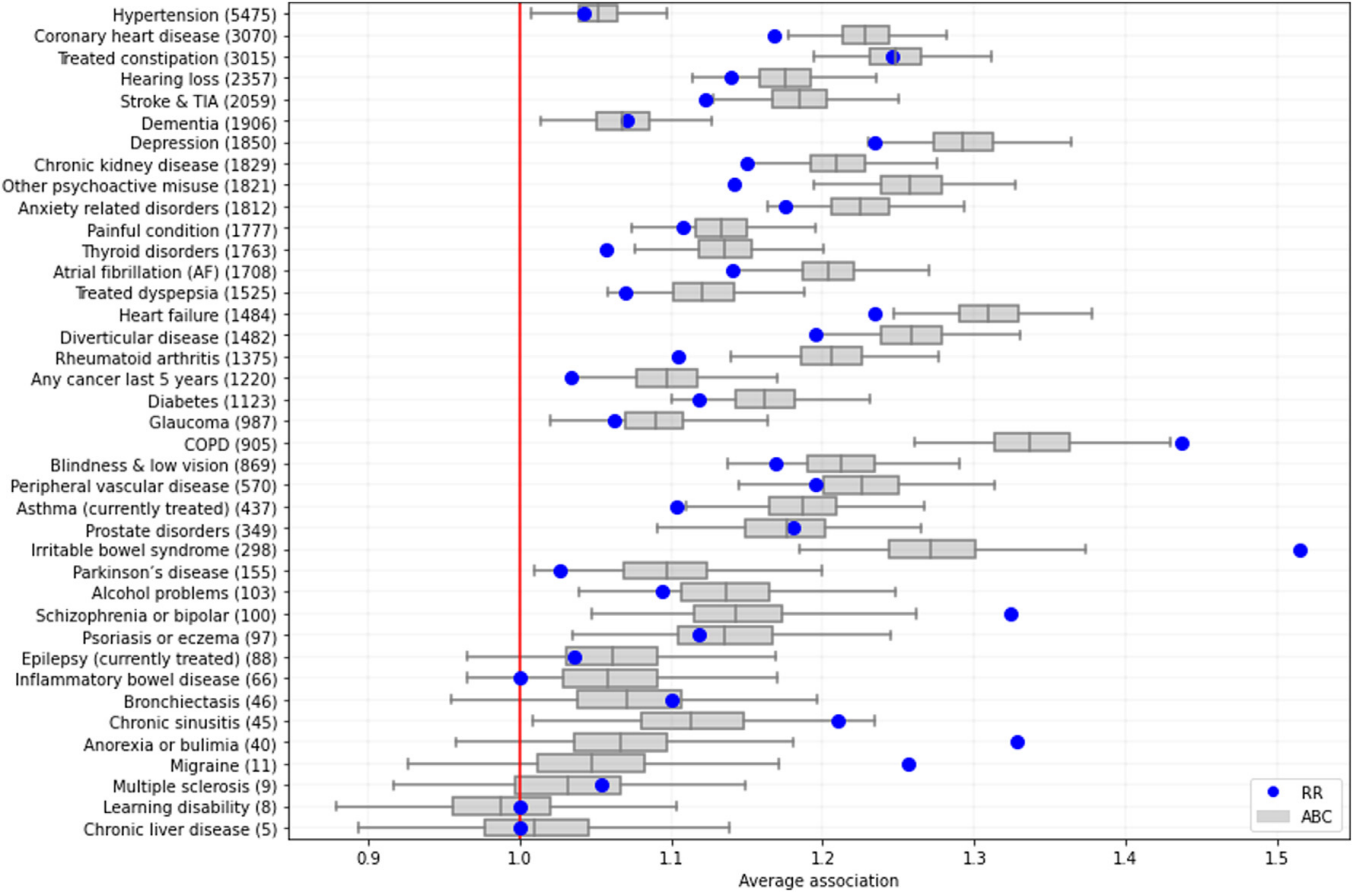
Average associations by ABC and RR of the 40 LTCs, sorted by their prevalence in the cohort. The red vertical line corresponds to a score of one, implying that on average there are no associations attached to this LTC. Boxes show the median and interquartile range of the distribution, and whiskers extend to 99% credible intervals. Blue circles denote average associations as computed by RR.

Last, we analysed the average association of each individual LTC to all others (Fig. 5). For RR (blue dots), the LTCs with the highest average association are *Irritable bowel syndrome* (1.52), *COPD* (1.44), *Anorexia or bulimia* (1.33), and *Schizophrenia, other psychosis or bipolar* (1.32). For ABC (boxplots), the LTCs with the strongest average association are *COPD* (1.34 [99% CI 1.25–1.44]), *Heart failure* (1.31 [99% CI 1.24–1.38]), *Depression* (1.29 [99% CI 1.23–1.37]), and *Irritable Bowel Syndrome* (1.27 [99% CI 1.18–1.39]). Average associations estimated by RR are in many cases within the credible intervals of ABC estimations or slightly below, with the notable exception of the top five average associations by RR: *Irritable bowel syndrome*, *COPD*, *Schizophrenia, other psychosis or bipolar*, *Anorexia or bulimia*, and *Migraine*. Most of these correspond to infrequent conditions where their high average RR associations rely on only a few significant associations with remarkably high RR values but moderate ABC values. For instance, the average RR association for *Migraine* only includes a single significant association by RR (with *Irritable Bowel Syndrome* [RR = 11.0, ABC = 1.3]) and *Anorexia or bulimia o*nly includes three significant associations by RR (with *Schizophrenia, other psychosis or bipolar* [RR = 9.0, ABC = 1.3], *Peripheral vascular disease* [RR = 3.7, ABC = 1.5], and *Blindness* [RR = 3.1, ABC = 1.6]). When stratifying by sex, we found a considerable disagreement between the top RR and ABC average associations in women, where RR found high average associations for *Alcohol problems* (2.42) and *Learning disability* (2.42), whereas ABC found average associations below 1.20 for both LTCs (Supplementary Figure S12). When nonparametric bootstrap is used to obtain RR average associations, low-prevalence conditions receive higher values along with extremely high uncertainty, limiting the usefulness of the measure for this purpose (Supplementary Figure S14).

## Discussion

We have developed a statistically robust framework to analyse associations in multimorbidity data while providing explicit quantification of uncertainty and applied it to analyse multimorbidity patterns in the oldest old (>90 years old, and stratified by sex) in a representative population of Scotland. We have demonstrated important differences in interpretation compared to RR. Our ABC approach is more cautious in attributing association than RR; almost all associations found by ABC were also found by RR, but the reverse is not true, only 60–85% of the associations found by RR were considered significant by ABC. Most of these discrepancies occurred for low-prevalence conditions with low count rates which, remarkably, were given among the strongest association values by RR. The more conservative behaviour was also found when determining differences in associations between sexes, as only 17.9% of the associations found in both sexes were estimated significant by ABC compared to 59% by RR. Additional discrepancies between the approaches were found when integrating associations into network representations or average associations of individual LTCs. ABC networks presented clusters with a clearer structure than RR networks. Average associations of individual LTCs were mostly similar between both approaches, with a few exceptions corresponding to uncommon conditions, where average associations by RR were significantly higher than those by ABC.

The top associations found by RR cannot be easily linked to previous medical findings. For instance, RR reported that the strongest association in men was between *Peripheral vascular disease* and *Anorexia or bulimia,* while in women was between *Alcohol problems* and *Learning disabilities*. Neither of these pairs was found to be significantly associated using ABC. While these associations may occur beyond chance, there is limited evidence in the wider literature or from mechanistic studies to support these as the most strongly linked conditions in our panel of LTCs. Conversely, the top associations found by our ABC approach were between *Depression* and *Anxiety-related disorders* in men, and between *COPD* and *Active asthma* in women. These LTCs are well-recognised to have a high association, even if the latter example may arise in part due to diagnostic uncertainty.

Regarding negative associations found by both methods, it stands out that several of them involve dementia, particularly in women. This may be an effect of age and survivorship bias, as individuals with a higher multimorbidity burden, particularly for cardiovascular conditions, may die from these conditions earlier, leaving apparent independence of these conditions for the oldest old population with dementia, which has much higher chances of appearing on survivors to later ages (Supplementary Figure S13). Therapeutic nihilism is also well recognised in older people and particularly in those with dementia, which might reduce the likelihood of comorbidity identification in this dataset where some conditions are ascertained using prescription data (for example, *Painful condition*, which is negatively associated with *Dementia* and where pain is known to be under-recognised).^24^^,^^25^

Regarding the aggregated measures of multimorbidity (association networks and average associations), several conditions with low or minimal representation in the dataset are in pairs with the strongest observed relative risk (such as *Migraine*, *Anorexia or bulimia*, *Chronic sinusitis*, *Schizophrenia, other psychosis or bipolar*, and *Irritable bowel syndrome*) strongly affecting these aggregated outcomes (Figs. 4 and 5) but there is little obvious reason to believe them to be so strongly associated from a clinical perspective. There might of course be undiscovered biological mechanisms which link these pairs, but it is very unlikely for example that such a strong biologically based association between schizophrenia or bipolar disease and anorexia or bulimia would not have been observed in younger people where both are much more common. We believe the more conservative ABC measure of association more appropriately draws attention to more plausible groups (for example, the more plausible clustering of cardiovascular and respiratory diseases in the ABC network of Fig. 4A vs the clusters found in the RR network of Fig. 4B).

We note that these discrepancies in results among methods mostly occur for associations related to infrequent LTCs. Indeed, strong associations by RR can be seen between pairs of LTCs for which count rates are low, such as *Learning disability*, *Migraine*, and *Anorexia or bulimia*. This is not surprising since RR has been previously reported to overestimate associations between pairs of infrequent conditions,^6^^,^^10^ amplifying the effects of inaccuracies in the data and increasing the susceptibility of the model to spurious findings.^4^^,^^22^ Moreover, although standard methods such as RR allow testing for statistical significance, they rely on assumptions (such as convergence to large-sample normality) that do not necessarily hold for associations between infrequent LTCs for which there are few observations.^4^^,^^14^^,^^22^ In contrast, in the presence of low counts due to low prevalence rates, the ABC approach is appropriately more cautious in attributing association for low prevalence conditions. A reason for this is that the notion of prior belief in the Bayesian approach counters the overestimation of associations between uncommon LTCs when less data are available and ensures robustness against arbitrary changes to significance thresholds. Although this caution is desirable in a holistic study of multimorbidity, it may also introduce some bias against rare true positives if the goal is to discover single new associations, and we recommend contrasting both methods in these contexts. Another important aspect of ABC is that it is a model-based measure using a joint model, so it does not suffer from problems coming from using p-values to test multiple comparisons while it also mitigates some of the confounding effects behind purely pairwise measures such as RR. Last, the statistical distributions used to evaluate significance with ABC stem from a generative model that explicitly defines uncertainty in the estimations, including for rare conditions, dispensing with approximations.^17^

A strength of our study is that we used a large dataset of Electronic Health Records from a representative population in Scotland. For this dataset, we had access to a previously curated set of LTCs, including physical and mental conditions whose definition accounted for drug prescriptions besides direct health record coding.^19^ The large size of the dataset provided enough numbers in the oldest old cohort, which was employed as a target cohort whose data availability is limited and hence typically excluded from research. Our methodological approach is also applicable to any analysis of small groups of people, including other population subgroups who may be infrequent even in large studies, such as ethnic minorities.

A limitation of our study lies in the difficulty of determining with certainty whether discrepancies in positive reporting truly are false positives, due to a lack of a ‘gold standard’ in the studied population. We have provided clinical interpretation to some of these, but future research is needed to provide further certainty about the nature of these discrepancies. One possibility is to analyse them in patients of younger age with larger numbers, although some associations may be particular to the oldest old cohort or not be present in it due to the survivorship effect. Therefore, this comparison should only be made in associations expected to appear much earlier in life, such as *Schizophrenia or bipolar disorder* and *Anorexia or bulimia*. These two conditions had an association value of RR = 9.0 [3.81–21.3], while it was not deemed significant by ABC. However, if we extend the cohort to include individuals 85 years old and older, RR also considers this association as not significant, suggesting that it may be a false positive and showing the instability of the RR measure (Supplementary Table S13). Other venues to validate the associations can be to examine the strength of genetic or molecular associations to explore which association measures are most effective for discovering underlying mechanisms or to perform experiments on synthetic data, and we leave this exercise for future work. Another limitation of our study is the use of cross-sectional data, without accounting for the chronological appearance of conditions. Including the temporal dimension would lead to a richer analysis as associations could be granted a direction, providing stronger hints towards causality.^14^^,^^26^ Future research would also be further enriched with a more fine-grained definition of health conditions, as a broad categorisation risks merging conditions with distinct pathophysiological mechanisms. From the modelling perspective, it would be of interest to explicitly include relevant factors such as socio-demographic characteristics. This would allow for instance to isolate the effect of the survivorship bias. Additionally, we have focused on RR as a standard association measure in the study of multimorbidity.^6^^,^^7^^,^^14^^,^^27^, ^28^, ^29^ However, a few other measures have been used, such as Pearson's φ-correlations,^5^^,^^7^^,^^11^ odds ratios^,^^7^^,^^15^^,^^16^ or the Salton Cosine Index.^7^^,^^30^ These share many of the problems of RR, such as bias towards conditions of specific prevalence or problems with low count rates.^4^^,^^6^^,^^7^ Still, these problems may affect their association results differently, and it would be of interest to analyse them rigorously.

In this study, we have focused on the effect of low condition counts on association measures and the importance of a sound definition of uncertainty, an issue that has not been assessed in previous studies of multimorbidity. These problems are not only important in determining the significance of an association but also in performing more complex analyses on them, such as comparisons between subgroups or the aggregation of associations into broader multimorbidity measures. Crucially, comparisons between subgroups can only be meaningful if estimations of uncertainty are properly defined and appropriate statistical tests are performed on their distributions.^8^ To illustrate this, we have performed statistical tests to compare associations with RR between men and women, which is already beyond the standard in previous literature on associations between morbidities, which limits reports of comparisons to single numbers.^5^^,^^30^ Furthermore, over-estimations of potentially spurious associations get amplified when analysing the complex interactions between LTCs, as false positives in specific associations compound when aggregated into broader measures of multimorbidity, such as average associations, node degree distributions, or morbidity clusters.^17^ Our approach allows us to perform principled statistical comparisons of these measures between subgroups too, as we can quantify their uncertainty instead of providing single-point estimates.

Our proposed method to find statistically robust associations beyond chance could advance the uncovering of common underlying mechanisms between health conditions. Because our method is based on a statistically robust Bayesian framework, it increases the reliability of multimorbidity research in underrepresented populations for which fewer data are available.

## Contributors

GRM, VR, and JF contributed to the study concept and design. GRM did the analysis, developed the software, interpreted the data, produced the figures, and wrote the original draft, with assistance from VR and JF, who supervised the work. GRM, JF and BG had access to and verified the underlying study data. AA helped with the medical literature search and AA, BG, and SWM provided medical feedback on the methods, analysis and interpretation of the results. BG, JF, and VR led the project administration. BG, JF, VR, SWM, and AA obtained funding. All authors reviewed and interpreted the results, commented on the paper, contributed to revisions, and read and approved the final version.

## Data sharing statement

Summary-level data of all associations are provided in the Supplementary Table S2. Code for performing the experiments will be available on GitHub (https://github.com/Juillermo/ABC) upon publication.

## Declaration of interests

We declare that we have no conflicts of interest.
