# Supplementary material for: Multimorbidity analysis with low condition counts: a robust Bayesian approach for small but important subgroups
Source: eBioMedicine. 2024 Mar 21;102:105081. doi: 10.1016/j.ebiom.2024.105081 (PMC10966445; doi:10.1016/j.ebiom.2024.105081)
Supplement: Supplementary material [file mmc1.pdf]

Contents

1 Supplementary methods **1**
1.1 Methods for finding associations between health conditions 1
1.2 Generative model of Associations Beyond Chance 1
1.3 Bayesian inference of ABC from Electronic Health Records 2
1.4 Determining the highest posterior density from posterior samples 3
1.5 Determining significance and confidence intervals with RR 4
1.6 Computing the overlap between two distributions 4
1.7 Computing average associations of an LTC with ABC and RR 4

2 Supplementary results **5**
2.1 Results stratified by sex 19

1 Supplementary methods

1.1 Methods for finding associations between health conditions

Previous studies on multimorbidity have used several different measures of association for building network representations, with the most popular being Relative Risk (RR) and Pearson's ϕ-correlations [1]. These measures are known to have biases in favour of rare conditions (RR) or against pairs including a rare and a frequent condition (ϕ-correlation) [2, 3]. RR presents the advantage of an easier interpretation by medical experts, while ϕ-correlation values are not straightforward to interpret, as its range of possible values depends on the prevalence of the conditions in the pair and is often much narrower than the theoretical limit of $[-1, 1]$ (achievable only when both conditions have a prevalence of 0.5) [2]. Other less frequently used measures include the Salton Cosine Index [4], odds ratios [5], and conditional probabilities [6, 7].

Another strand of literature employs Bayesian Networks (BNs) [8] to find a causal structure between the conditions. This methodology uses Directed Acyclic Graphs (DAGs), where all connections have a direction (of causality) and loops are not allowed, so mechanisms between conditions working in different directions cannot be properly captured. Additionally, in this framework, each link has an associated probability of existence, with results typically falling into two modes around zero and one, hence effectively providing binary information and no association strength.

Furthermore, the understanding of multimorbidity can also be approached from clustering methods, which aim to build profiles of patients within a population to identify typical combinations of long–term conditions [9, 10]. Although this approach can be useful for improving the management of multimorbid patients by incorporating these profiles into the design of healthcare pathways, it provides weaker aetiological signals. Half-way between pairwise associations and clusters of patients, dimensionality reduction techniques, such as Factor Analysis or Principal Component Analysis [9, 10], build latent representations that may guide the grouping of conditions. A post-hoc analysis of the latent representations can help build hypotheses about the aetiological factors, but the focus is on a broad grouping of conditions, unlike pairwise approaches that reveal fine–grained relationships between conditions [11].

1.2 Generative model of Associations Beyond Chance

Given a set of LTCs, we define the appearance of each LTC on a patient by a random binary variable, $LTC_i \in \{0, 1\}$, $i \in I = 1, \ldots, M$. Our model assumes that the appearance of these LTCs can be due to hypothetical *independent risk factors* f_i that affect the appearance of each LTC independently, and hypothetical common mechanisms f_{ij} that affect a pair of LTCs simultaneously. This model is similar in spirit to that of [12], where they model the appearance of diseases as generated by the accumulation of deleterious mutations in three disjoint sets of genes: those affecting both diseases simultaneously and those affecting each disease independently. Note that this modelling approach dismisses the explicit modelling of higher–order associations (i.e. mechanisms f_{ijk} affecting three LTCs simultaneously, f_{ijkl} affecting four, etc).

According to this model, the joint probabilities of a patient having the pair LTC_i and LTC_j as

$$\begin{cases} \mathbb{P}(LTC_i = 1, LTC_j = 1 | f_i, f_j, f_{ij}) = & f_{ij} + f_i f_j - f_{ij} f_i f_j \\ \mathbb{P}(LTC_i = 1, LTC_j = 0 | f_i, f_j, f_{ij}) = & f_i(1 - f_j)(1 - f_{ij}) \\ \mathbb{P}(LTC_i = 0, LTC_j = 1 | f_i, f_j, f_{ij}) = & f_j(1 - f_i)(1 - f_{ij}) \\ \mathbb{P}(LTC_i = 0, LTC_j = 0 | f_i, f_j, f_{ij}) = & (1 - f_i)(1 - f_j)(1 - f_{ij}) \end{cases}, \quad (1)$$

where equations in (1) have been chosen to simulate conditionally independent factors. To ensure that probabilities remain within the $[0,1]$ bound, we restrict independent risk factors to $f_i \in [0,1]$ and common mechanisms to $f_{ij} \in [-f_i f_j/(1-f_i f_j), 1]$.

We then define *Association Beyond Chance* ABC_{ij} measure between $LTCi$ and $LTCj$ as the ratio between the contribution to their co-appearance coming from common mechanisms f_{ij} to the contribution coming from independent factors. For a direct comparison with relative risk (observed–to–expected ratio), we set the independence value at $ABC^{(ind)} = 1$,

$$ABC_{ij} = 1 + \frac{f_{ij}}{f_i f_j} \ . \tag{2}$$

And independence value of $ABC^{(ind)} = 1$ is then achieved when $f_{ij} = 0$, implying that there is no common mechanism that links both LTCs and their co-occurrence may happen exclusively by chance from each LTC occurring due to their independent risk factors f_i. Values $ABC > 1$ (*positive* associations) suggest the presence of common mechanisms that increase the co-occurrence of both LTCs, while values $ABC < 1$ (*negative* associations) suggest the presence of mechanisms that hinder the co-occurrence of the LTCs. Note that ABC is defined within the range $ABC_{ij} \in [-f_i f_j/(1-f_i f_j), \infty)$. To induce symmetry between positive and negative associations, it is useful to examine $\log ABC_{ij}$ (with a cut-off at $ABC_{ij} = 0$) instead of ABC_{ij} directly.

Bayesian inference of our model requires choosing prior distributions on the parameters. Selecting a prior in the association value ABC_{ij} is more intuitive than in the common mechanism f_{ij}, and therefore we set priors $\mathbb{P}(ABC_{ij})$ and $\mathbb{P}(f_i)$ instead of $\mathbb{P}(f_{ij})$ and $\mathbb{P}(f_i)$. Note that we are not assuming complex correlations between the prior distributions and keep each prior independent, although we do assume that all parameters ABC_{ij} are generated by the same prior distribution, as well as all parameters f_i.

Since the values of ABC_{ij} are only lower–bounded, we choose a log-normal distribution for their generation, parameterised by the mean μ and standard deviation σ of the normal distribution in the logarithmic scale. As independent risk factors are restricted to the interval $[0,1]$, we use beta distributions for their generation, parameterised by α and β.

We need to choose the values of these distribution parameters, $\Theta = \{\mu, \sigma, \alpha, \beta\}$. To prevent our model from reporting false positives, we set $\mu = 0$, which gives priority to the outcome of 'no association' when evidence is limited. Although the choice of values for the other parameters in Θ could be based on medical knowledge, we used a non-informative approach by setting *hyperprior* distributions for each of them, allowing the inference process to find appropriate values based on the data and freeing our model from potential bias from previous knowledge. As σ, α, β are all unbounded positive numbers, we assume exponential distributions for their generation, with $\lambda_\alpha = \lambda_\beta = 0.01$ and $\lambda_\sigma = 1$.

In summary, our priors are defined by

$$\begin{array}{ccc} ABC_{ij} & \sim & \mathrm{Log-normal}(\mu = 0, \sigma) \ , \\ \sigma & \sim & \mathrm{Exponential}(\lambda_\sigma = 1) \ , \\ f_i & \sim & \mathrm{Beta}(\alpha, \beta) \ , \\ \alpha, \beta & \sim & \mathrm{Exponential}(\lambda_\alpha = \lambda_\beta = 0.01) \ . \end{array} \tag{3}$$

A diagram showing the generative model here defined can be seen in Fig. 1.

1.3 Bayesian inference of ABC from Electronic Health Records

We assume a cross-sectional dataset D of Electronic Health Records with N patients and M LTCs, where each patient has a combination of LTCs $D_n \in \{0,1\}^M$, $n = 1, \ldots, N$. Since our model, as defined in Eq. (1), is based on pairwise associations, the combinations of co-occurrences $X = (X_{ij})$ between pairs of LTCs and the marginal counts $\boldsymbol{P} = (P_i)$ of each LTC are sufficient statistics for our inference problem. We assume that the appearance of LTCs in different patients is independent and identically distributed, and therefore the probability of observing a specific combination $\{X_{ij}, P_i, P_{ij}\}$ is given by the multinomial distribution

$$\mathbb{P}(X_{ij}, P_i, P_j | f_i, f_j, ABC_{ij}) = \frac{\Gamma(N+1)}{\prod_k \Gamma(N_k+1)} \prod_{k=1}^{4} p_k^{N_k} \ , \tag{4}$$

where

$$\begin{cases} k=1, & N_1 = X_{ij} \ , & p_1 = \mathbb{P}(LTC_i = 1, LTC_j = 1 | f_i, f_j, ABC_{ij}) \\ k=2, & N_2 = P_i - X_{ij} \ , & p_2 = \mathbb{P}(LTC_i = 1, LTC_j = 0 | f_i, f_j, ABC_{ij}) \\ k=3, & N_3 = P_j - X_{ij} \ , & p_3 = \mathbb{P}(LTC_i = 0, LTC_j = 1 | f_i, f_j, ABC_{ij}) \\ k=4, & N_4 = N - P_i - P_j - X_{ij} \ , & p_4 = \mathbb{P}(LTC_i = 0, LTC_j = 0 | f_i, f_j, ABC_{ij}) \end{cases} \ . \tag{5}$$

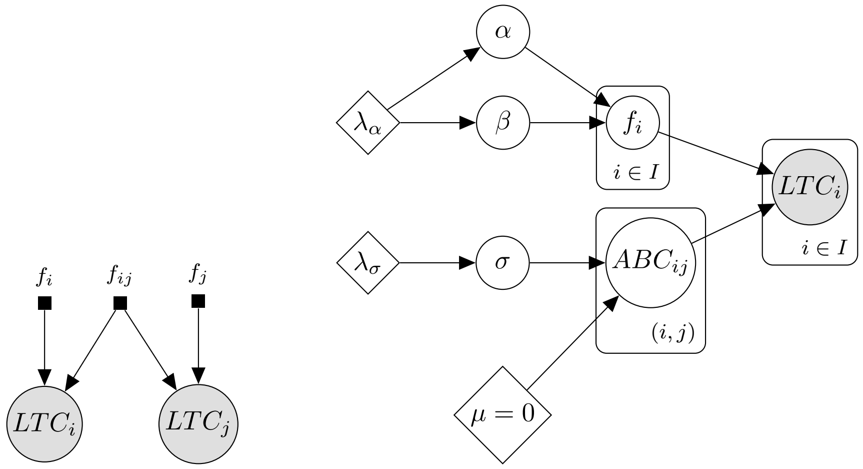

Figure 1: Left: Factor graph for the generation of a pair of LTCs. Right: Plate notation describing the Probabilistic Graphical Model for all LTCs. These graphs were generated with the TikZ library BayesNet.

Note that in Eq. (4) we have assumed that the probabilities of finding co-occurrences between pairs of conditions are *marginally independent* to finding co-occurrences in other pairs of conditions, such that

$$\mathbb{P}\left(X, \boldsymbol{P} | \boldsymbol{f}, F\right) = \prod_{(i,j)} \mathbb{P}\left(X_{ij}, P_i, P_j | f_i, f_j, ABC_{ij}\right) \ , \tag{6}$$

where $\boldsymbol{f}$ is the vector of all individual factors, ABC is the matrix aggregating all associations ABC_{ij}, and (i,j) are combinations over all pairs of LTCs. Although we factorise the probabilities of finding co-occurrences and prevalence counts, we constrain each f_i to be unique per LTC and shared across all pairwise associations, hence capturing some form of interaction with other pairwise associations.

Given the model above, we apply a Bayesian framework for the inference of the posterior distribution for all parameters f_i and ABC_{ij},

$$\mathbb{P}\left(ABC, \boldsymbol{f} | X, \boldsymbol{P}\right) = \frac{\mathbb{P}\left(X, \boldsymbol{P} | \boldsymbol{f}, ABC\right) \mathbb{P}\left(\boldsymbol{f}, ABC\right)}{\mathbb{P}\left(X, \boldsymbol{P}\right)} \ , \tag{7}$$

Where the priors $\mathbb{P}\left(\boldsymbol{f}, ABC\right)$ are defined by

$$\mathbb{P}\left(ABC, \boldsymbol{f}\right) = \mathbb{P}\left(ABC_{ij} | \mu, \sigma\right)^{\binom{M}{2}} \mathbb{P}\left(\sigma | \lambda_\sigma\right)^{\binom{M}{2}} \mathbb{P}\left(f_i | \alpha, \beta\right)^{M} \mathbb{P}\left(\alpha | \lambda_\alpha\right)^{M} \mathbb{P}\left(\beta | \lambda_\beta\right)^{M} \ , \tag{8}$$

where $\binom{M}{2} = M(M-1)/2$ is a binomial coefficient and the distributions of the priors are given in Eq. (3).

Since the computation of the denominator in Eq. (7) requires the integration over all variables, which is computationally infeasible, we resort to Hamiltonian Monte Carlo (HMC) techniques to obtain samples of the parameters from the posterior distribution, which we implemented using the statistical library *Stan* [13]. For each inference result, we ran five chains of the HMC algorithm with 500 warm-up samples that get discarded and $2 \cdot 10^4$ samples from the posterior distributions. Code for running the experiments can be found at `https://github.com/Juillermo/ABC`.

1.4 Determining the highest posterior density from posterior samples

For reporting association values, summary statistics of the posterior distribution such as the mean or median can be misleading, as these distributions may be highly skewed, having long tails. A better statistic is the Highest Posterior Density (HPD) interval, which corresponds to the mode of the posterior distribution. As our posterior distribution is characterised by a cloud of samples, an approximation to the HPD is to take the region of the parameter space where the density of samples is the highest. To achieve this, we sorted the samples of the variable we wanted to summarise and divided them into 100 groups, with 200 samples each. We then selected the group with the shortest span (i.e., the difference between the highest and lowest samples) and reported the mid-range of the span.

1.5 Determining significance and confidence intervals with RR

For determining the significance of an association between two LTCs by RR, we used Fisher's exact test, which is robust to low cell counts (infrequent LTCs) [2]. We obtained confidence intervals for RR via the Katz method, which assumes RR follows a normal distribution in the log-scale with mean $\mu = RR$ and standard deviation $\sigma = 1/X_{ij} - 1/N + 1/P_iP_j - 1/N^2$. A 99% confidence interval was then retrieved with $[RR \exp(\pm 2.56\sigma)]$ [14, 2].

1.6 Computing the overlap between two distributions

To assess the differences between two distributions, we computed the overlap between them. Distributions from the Bayesian inference model are defined by sets of samples, so we estimated their overlap by looking at the probability that samples from one of the distributions are higher than samples from the other, i.e., $\mathbb{P}(D_1 > D_2)$. Probabilities values close to 0 or 1 imply that one of the distributions is consistently higher than the other and hence there is little overlap, so we defined the p-value of them being different as $p = \min[\mathbb{P}(D_1 > D_2), \mathbb{P}(D_2 > D_1)]$.

For RR, we used the Katz approximation that assumes RR follows a normal distribution in the log-scale with mean $\mu = RR$ and $\sigma = 1/X_{ij} - 1/N + 1/P_iP_j - 1/N^2$, computed the intersection of the two normal distributions, and defined the p-value as the integral of the area of overlap between them.

1.7 Computing average associations of an LTC with ABC and RR

The computation of an LTC's average association varied from results with ABC and RR, as these two quantities are mathematically different.

For ABC, we obtained average associations by computing $ABC_i = 1/M \sum_j^M ABC_{ij}$ for each sample of the posterior, hence providing a distribution of ABC_i values that reflects the uncertainty in the measure. We included both significant and non-significant associations for this computation, as the latter can still propagate uncertainty into the measures.

For RR, we counted non-significant associations as $RR = 1$, with average associations computed as $RR_i = 1/M \left(\sum_{j \in S} RR_{ij} + |\bar{S}|\right)$, where $|\bar{S}|$ is the number of non-significant associations. As each individual RR_{ij} is modelled as a random variable with log-normal distribution, the resulting distribution for RR_i is a sum of log-normal distributions, with no exact functional form known. Although various approximations for this distribution have been proposed (e.g. Fenton–Wilkinson), it is still not straightforward to combine the information of non-significant associations into this computation from a parametric perspective. For simplicity, we rely on central estimates for average associations with RR, although we also provide results for RR with non-parametric bootstrap in Supplementary Figure S14.

2 Supplementary results

	Total (N=12009)	Men (N=3039)	Women (N=8970)
Hypertension	5475 (46%)	1142 (38%)	4333 (48%)
Coronary heart disease	3070 (26%)	900 (30%)	2170 (24%)
Treated constipation	3015 (25%)	672 (22%)	2343 (26%)
Hearing loss	2357 (20%)	646 (21%)	1711 (19%)
Stroke & transient ischaemic attack (TIA)	2059 (17%)	568 (19%)	1491 (17%)
Dementia	1906 (16%)	310 (10%)	1596 (18%)
Depression	1850 (15%)	299 (9.8%)	1551 (17%)
Chronic kidney disease (CKD)	1829 (15%)	454 (15%)	1375 (15%)
Other psychoactive misuse	1821 (15%)	349 (11%)	1472 (16%)
Anxiety & other neurotic, stress related & somatic disorders	1812 (15%)	271 (8.9%)	1541 (17%)
Painful condition	1777 (15%)	309 (10%)	1468 (16%)
Thyroid disorders	1763 (15%)	206 (6.8%)	1557 (17%)
Atrial fibrillation (AF)	1708 (14%)	472 (16%)	1236 (14%)
Treated dyspepsia	1525 (13%)	306 (10%)	1219 (14%)
Heart failure	1484 (12%)	410 (13%)	1074 (12%)
Diverticular disease of intestine	1482 (12%)	311 (10%)	1171 (13%)
Rheumatoid arthritis, other inflammatory polyarthropathies & systematic connective tissue disorders	1375 (11%)	319 (10%)	1056 (12%)
New diagnosis of cancer in last five years	1220 (10%)	399 (13%)	821 (9.2%)
Diabetes	1123 (9.4%)	321 (11%)	802 (8.9%)
Glaucoma	987 (8.2%)	290 (9.5%)	697 (7.8%)
Chronic obstructive pulmonary disease (COPD)	905 (7.5%)	343 (11%)	562 (6.3%)
Blindness & low vision	869 (7.2%)	199 (6.5%)	670 (7.5%)
Peripheral vascular disease	570 (4.7%)	190 (6.3%)	380 (4.2%)
Asthma (currently treated)	437 (3.6%)	104 (3.4%)	333 (3.7%)
Prostate disorders	349 (2.9%)	349 (11%)	0 (0%)
Irritable bowel syndrome (IBS)	298 (2.5%)	49 (1.6%)	249 (2.8%)
Parkinson's disease	155 (1.3%)	59 (1.9%)	96 (1.1%)
Alcohol problems	103 (0.86%)	49 (1.6%)	54 (0.6%)
Schizophrenia (and related non-organic psychosis) or bipolar disorders	100 (0.83%)	18 (0.59%)	82 (0.91%)
Psoriasis or eczema	97 (0.81%)	38 (1.3%)	59 (0.66%)
Epilepsy (currently treated)	88 (0.73%)	38 (1.3%)	50 (0.56%)
Inflammatory bowel disease	66 (0.55%)	11 (0.36%)	55 (0.61%)
Bronchiectasis	46 (0.38%)	11 (0.36%)	35 (0.39%)
Chronic sinusitis	45 (0.37%)	10 (0.33%)	35 (0.39%)
Anorexia or Bulimia	40 (0.33%)	7 (0.23%)	33 (0.37%)
Migraine	11 (0.092%)	< 5(< 0.2%)	9 (0.1%)
Multiple sclerosis	9 (0.075%)	0 (0%)	9 (0.1%)
Learning disability	8 (0.067%)	< 5(< 0.2%)	6 (0.067%)
Chronic liver disease	5 (0.042%)	< 5(< 0.2%)	< 5(< 0.06%)
Viral Hepatitis	< 5(< 0.04%)	< 5(< 0.2%)	0 (0%)

Table 1: **Set of studied LTCs and their prevalence in the oldest–old cohort.** Counts and prevalence of LTCs in the oldest–old population (90 years old and above). Conditions are ordered by their prevalence in the whole cohort.

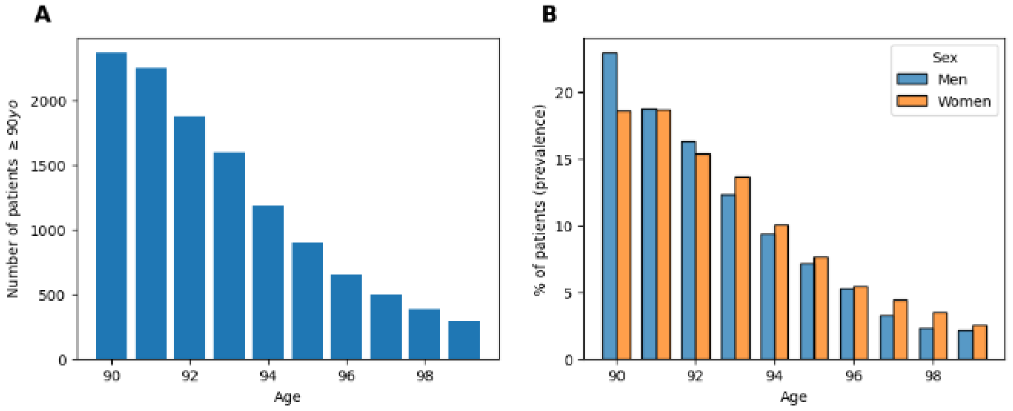

Figure 2: **Age distribution in our oldest–old cohort in total (A) and stratified by sex (B).**

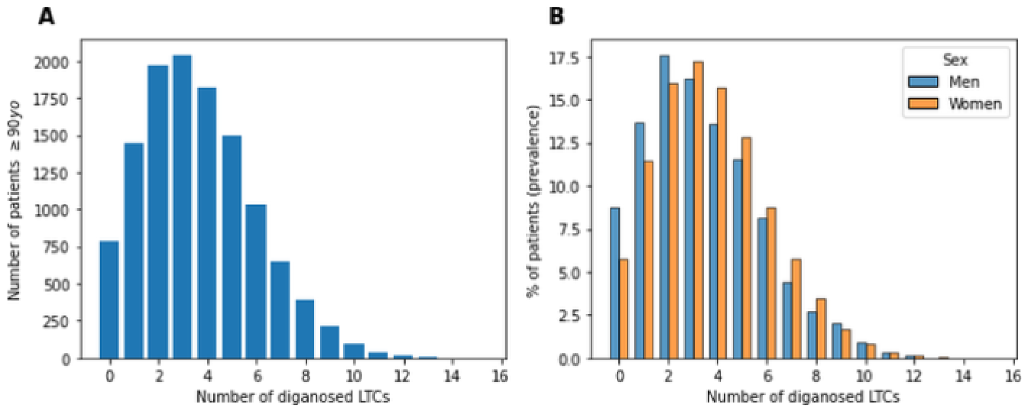

Figure 3: **Distributions of diagnosed LTCs in our oldest–old cohort in total (A) and stratified by sex (B).**

Table 2: Significant associations found by relative risk (RR) or Associations Beyond Chance (ABC) in our oldest–old cohort, sorted by RR values in descending order. 'NS' marks an association not deemed to be significant by the method.

	RR (CI 99%)	ABC (CI 99%)
Irritable bowel syndrome - Migraine	11.0 (4.65 - 26.0)	NS
Schizophrenia or bipolar - Anorexia or Bulimia	9.01 (3.81 - 21.3)	NS
Irritable bowel syndrome - Chronic sinusitis	5.37 (3.5 - 8.25)	NS
COPD - Bronchiectasis	4.9 (4.22 - 5.71)	2.38 (1.24 - 4.24)
Prostate disorders - Psoriasis or eczema	4.26 (3.44 - 5.28)	NS
Asthma (currently treated) - COPD	3.86 (3.78 - 3.93)	3.54 (2.87 - 4.43)
Peripheral vascular disease - Anorexia or Bulimia	3.69 (2.55 - 5.33)	NS
COPD - Chronic sinusitis	3.54 (2.86 - 4.39)	NS
Depression - Schizophrenia or bipolar	3.31 (3.15 - 3.48)	2.63 (1.89 - 3.84)
COPD - Alcohol problems	3.22 (2.91 - 3.57)	2.25 (1.28 - 3.4)
Blindness & low vision - Anorexia or Bulimia	3.11 (2.34 - 4.14)	NS
Treated constipation - Multiple sclerosis	3.1 (2.14 - 4.48)	NS
Irritable bowel syndrome - Diverticular disease	2.69 (2.62 - 2.76)	2.64 (1.94 - 3.25)
Depression - Anxiety related disorders	2.39 (2.38 - 2.39)	2.52 (2.25 - 2.76)
Atrial fibrillation (AF) - Heart failure	2.31 (2.3 - 2.32)	2.48 (2.15 - 2.7)
Glaucoma - Blindness & low vision	2.3 (2.26 - 2.33)	2.2 (1.82 - 2.71)
Hearing loss - Chronic sinusitis	2.26 (1.99 - 2.58)	1.71 (1.02 - 2.76)
Coronary heart disease - Heart failure	2.13 (2.12 - 2.14)	2.25 (2.05 - 2.45)
Chronic kidney disease - Heart failure	1.91 (1.9 - 1.92)	2.0 (1.77 - 2.26)

	RR (CI 99%)	ABC (CI 99%)
Dementia - Schizophrenia or bipolar	1.89 (1.74 - 2.06)	1.46 (1.03 - 2.43)
Treated dyspepsia - Irritable bowel syndrome	1.88 (1.81 - 1.95)	1.83 (1.3 - 2.35)
Depression - Dementia	1.87 (1.86 - 1.88)	1.88 (1.72 - 2.14)
COPD - Heart failure	1.87 (1.85 - 1.89)	1.87 (1.58 - 2.25)
COPD - Peripheral vascular disease	1.86 (1.8 - 1.92)	1.72 (1.34 - 2.35)
Stroke & TIA - Epilepsy (currently treated)	1.86 (1.69 - 2.03)	NS
Anxiety related disorders - Schizophrenia or bipolar	1.86 (1.69 - 2.03)	NS
Diabetes - Chronic kidney disease	1.84 (1.83 - 1.86)	1.88 (1.65 - 2.2)
Depression - Irritable bowel syndrome	1.83 (1.77 - 1.89)	1.75 (1.36 - 2.36)
COPD - Prostate disorders	1.83 (1.73 - 1.93)	1.65 (1.17 - 2.34)
Alcohol problems - Heart failure	1.81 (1.62 - 2.02)	NS
Any cancer last 5 years - Prostate disorders	1.78 (1.71 - 1.85)	1.63 (1.17 - 2.25)
Treated constipation - Psoriasis or eczema	1.77 (1.66 - 1.87)	1.53 (1.07 - 2.22)
Prostate disorders - Glaucoma	1.74 (1.66 - 1.83)	1.66 (1.12 - 2.18)
Anxiety related disorders - Irritable bowel syndrome	1.73 (1.68 - 1.79)	1.74 (1.27 - 2.2)
Depression - Treated constipation	1.73 (1.73 - 1.74)	1.83 (1.67 - 1.98)
Treated constipation - Parkinson's disease	1.7 (1.63 - 1.76)	1.58 (1.15 - 2.16)
Coronary heart disease - Other psychoactive misuse	1.69 (1.69 - 1.7)	1.79 (1.62 - 1.96)
Diabetes - Blindness & low vision	1.69 (1.65 - 1.72)	1.64 (1.36 - 2.1)
Irritable bowel syndrome - Treated constipation	1.68 (1.65 - 1.72)	1.62 (1.34 - 2.04)
Peripheral vascular disease - Heart failure	1.68 (1.64 - 1.71)	1.65 (1.36 - 2.1)
Coronary heart disease - Peripheral vascular disease	1.66 (1.64 - 1.68)	1.66 (1.44 - 1.98)
Alcohol problems - Dementia	1.65 (1.5 - 1.82)	NS
Anxiety related disorders - Treated constipation	1.65 (1.64 - 1.65)	1.74 (1.58 - 1.87)
Rheumatoid arthritis - Heart failure	1.63 (1.62 - 1.65)	1.71 (1.45 - 1.97)
Dementia - Parkinson's disease	1.63 (1.52 - 1.73)	1.49 (1.03 - 2.07)
Depression - Painful condition	1.63 (1.62 - 1.63)	1.69 (1.49 - 1.9)
Painful condition - Treated constipation	1.61 (1.6 - 1.61)	1.67 (1.53 - 1.83)
Diabetes - Other psychoactive misuse	1.6 (1.59 - 1.62)	1.66 (1.4 - 1.94)
Stroke & TIA - Atrial fibrillation (AF)	1.6 (1.59 - 1.61)	1.7 (1.48 - 1.85)
Hearing loss - Psoriasis or eczema	1.58 (1.45 - 1.72)	NS
Treated constipation - Schizophrenia or bipolar	1.55 (1.45 - 1.66)	NS
Depression - Parkinson's disease	1.55 (1.45 - 1.66)	NS
Painful condition - Irritable bowel syndrome	1.54 (1.49 - 1.6)	1.43 (1.11 - 2.02)
Treated constipation - Epilepsy (currently treated)	1.54 (1.43 - 1.66)	NS
Coronary heart disease - Chronic kidney disease	1.53 (1.52 - 1.53)	1.61 (1.47 - 1.76)
Coronary heart disease - Atrial fibrillation (AF)	1.5 (1.5 - 1.51)	1.59 (1.43 - 1.74)
Depression - Treated dyspepsia	1.5 (1.49 - 1.51)	1.5 (1.34 - 1.77)
COPD - Diverticular disease	1.5 (1.47 - 1.52)	1.48 (1.25 - 1.83)
Diabetes - Heart failure	1.49 (1.47 - 1.51)	1.53 (1.29 - 1.83)
Rheumatoid arthritis - Peripheral vascular disease	1.49 (1.45 - 1.53)	1.49 (1.15 - 1.9)
Painful condition - Rheumatoid arthritis	1.48 (1.47 - 1.5)	1.51 (1.31 - 1.76)
Chronic kidney disease - Atrial fibrillation (AF)	1.48 (1.47 - 1.49)	1.54 (1.35 - 1.74)
Painful condition - Treated dyspepsia	1.48 (1.46 - 1.49)	1.55 (1.28 - 1.73)
Stroke & TIA - Peripheral vascular disease	1.47 (1.45 - 1.5)	1.49 (1.21 - 1.85)
Treated constipation - Dementia	1.46 (1.46 - 1.47)	1.52 (1.37 - 1.66)
Heart failure - Prostate disorders	1.46 (1.4 - 1.52)	1.36 (1.05 - 1.89)
Painful condition - Anxiety related disorders	1.45 (1.44 - 1.46)	1.51 (1.31 - 1.7)
Other psychoactive misuse - Blindness & low vision	1.45 (1.43 - 1.47)	1.49 (1.21 - 1.78)
Other psychoactive misuse - Dementia	1.44 (1.43 - 1.45)	1.49 (1.3 - 1.67)
Treated dyspepsia - Anxiety related disorders	1.44 (1.43 - 1.45)	1.48 (1.28 - 1.67)
Rheumatoid arthritis - Diverticular disease	1.43 (1.42 - 1.45)	1.46 (1.26 - 1.71)
Anxiety related disorders - Diverticular disease	1.43 (1.42 - 1.44)	1.47 (1.29 - 1.7)
Coronary heart disease - Prostate disorders	1.42 (1.4 - 1.45)	1.43 (1.14 - 1.75)
Hypertension - Chronic kidney disease	1.42 (1.42 - 1.42)	1.48 (1.39 - 1.6)
Diverticular disease - Prostate disorders	1.42 (1.36 - 1.48)	NS
Asthma (currently treated) - Diverticular disease	1.41 (1.36 - 1.46)	1.48 (1.04 - 1.8)
Other psychoactive misuse - Heart failure	1.4 (1.39 - 1.42)	1.45 (1.28 - 1.69)
Hearing loss - Prostate disorders	1.4 (1.36 - 1.44)	1.38 (1.09 - 1.74)
Depression - Other psychoactive misuse	1.4 (1.39 - 1.41)	1.46 (1.29 - 1.66)

	RR (CI 99%)	ABC (CI 99%)
Other psychoactive misuse - Stroke & TIA	1.4 (1.39 - 1.41)	1.46 (1.28 - 1.65)
Treated dyspepsia - Treated constipation	1.39 (1.38 - 1.4)	1.42 (1.27 - 1.61)
Diabetes - Peripheral vascular disease	1.39 (1.34 - 1.44)	1.32 (1.02 - 1.84)
Treated dyspepsia - Diverticular disease	1.39 (1.37 - 1.4)	1.39 (1.2 - 1.65)
Atrial fibrillation (AF) - Peripheral vascular disease	1.38 (1.35 - 1.41)	1.37 (1.08 - 1.73)
Rheumatoid arthritis - Chronic kidney disease	1.38 (1.36 - 1.39)	1.43 (1.23 - 1.64)
Coronary heart disease - Diverticular disease	1.37 (1.37 - 1.38)	1.45 (1.25 - 1.61)
Chronic kidney disease - Peripheral vascular disease	1.37 (1.34 - 1.4)	1.39 (1.1 - 1.72)
Asthma (currently treated) - Heart failure	1.37 (1.32 - 1.42)	1.33 (1.03 - 1.78)
Atrial fibrillation (AF) - Prostate disorders	1.37 (1.32 - 1.42)	NS
Hearing loss - Irritable bowel syndrome	1.37 (1.32 - 1.41)	1.38 (1.03 - 1.77)
Other psychoactive misuse - Atrial fibrillation (AF)	1.37 (1.36 - 1.38)	1.45 (1.24 - 1.62)
Asthma (currently treated) - Anxiety related disorders	1.36 (1.33 - 1.4)	1.43 (1.03 - 1.78)
Hearing loss - COPD	1.36 (1.35 - 1.38)	1.4 (1.17 - 1.62)
Coronary heart disease - Diabetes	1.35 (1.34 - 1.36)	1.42 (1.23 - 1.59)
Diabetes - Stroke & TIA	1.35 (1.33 - 1.36)	1.37 (1.17 - 1.62)
Hearing loss - Diverticular disease	1.34 (1.34 - 1.35)	1.35 (1.22 - 1.58)
COPD - Atrial fibrillation (AF)	1.34 (1.32 - 1.36)	1.34 (1.13 - 1.64)
Coronary heart disease - Stroke & TIA	1.34 (1.33 - 1.34)	1.39 (1.26 - 1.54)
Diverticular disease - Blindness & low vision	1.33 (1.31 - 1.36)	1.34 (1.1 - 1.65)
Coronary heart disease - COPD	1.33 (1.32 - 1.34)	1.37 (1.19 - 1.58)
Rheumatoid arthritis - Hearing loss	1.33 (1.32 - 1.34)	1.38 (1.19 - 1.56)
Coronary heart disease - Rheumatoid arthritis	1.33 (1.32 - 1.33)	1.39 (1.22 - 1.55)
Stroke & TIA - Heart failure	1.32 (1.31 - 1.33)	1.41 (1.2 - 1.6)
Diabetes - Thyroid disorders	1.32 (1.3 - 1.33)	1.31 (1.12 - 1.58)
Hearing loss - Peripheral vascular disease	1.31 (1.29 - 1.34)	1.3 (1.09 - 1.67)
COPD - Blindness & low vision	1.31 (1.27 - 1.35)	1.31 (1.02 - 1.7)
Diabetes - Atrial fibrillation (AF)	1.31 (1.29 - 1.32)	1.34 (1.13 - 1.61)
Other psychoactive misuse - Treated constipation	1.31 (1.3 - 1.31)	1.34 (1.22 - 1.51)
COPD - Other psychoactive misuse	1.3 (1.29 - 1.32)	1.38 (1.1 - 1.62)
Hypertension - Peripheral vascular disease	1.3 (1.29 - 1.31)	1.31 (1.15 - 1.52)
Painful condition - Diverticular disease	1.3 (1.28 - 1.31)	1.35 (1.14 - 1.54)
Asthma (currently treated) - Chronic kidney disease	1.29 (1.25 - 1.33)	1.26 (1.01 - 1.69)
Thyroid disorders - Heart failure	1.29 (1.28 - 1.3)	1.35 (1.14 - 1.55)
Anxiety related disorders - Other psychoactive misuse	1.29 (1.28 - 1.3)	1.34 (1.18 - 1.55)
Treated constipation - Blindness & low vision	1.29 (1.28 - 1.3)	1.32 (1.15 - 1.54)
Diverticular disease - Heart failure	1.28 (1.27 - 1.3)	1.35 (1.14 - 1.57)
Rheumatoid arthritis - Blindness & low vision	1.28 (1.25 - 1.3)	1.26 (1.02 - 1.6)
Stroke & TIA - Diverticular disease	1.28 (1.27 - 1.29)	1.3 (1.15 - 1.53)
Depression - Thyroid disorders	1.27 (1.26 - 1.28)	1.32 (1.15 - 1.5)
Other psychoactive misuse - Chronic kidney disease	1.27 (1.26 - 1.28)	1.34 (1.15 - 1.52)
Hypertension - Diabetes	1.27 (1.27 - 1.28)	1.31 (1.18 - 1.45)
Rheumatoid arthritis - Atrial fibrillation (AF)	1.27 (1.26 - 1.28)	1.29 (1.11 - 1.55)
Depression - Diverticular disease	1.27 (1.25 - 1.28)	1.29 (1.13 - 1.53)
Thyroid disorders - Rheumatoid arthritis	1.26 (1.25 - 1.28)	1.29 (1.1 - 1.52)
Any cancer last 5 years - Diverticular disease	1.26 (1.24 - 1.27)	1.26 (1.07 - 1.52)
Treated constipation - Diverticular disease	1.26 (1.25 - 1.26)	1.3 (1.17 - 1.46)
Rheumatoid arthritis - COPD	1.25 (1.23 - 1.28)	1.3 (1.02 - 1.56)
Painful condition - Other psychoactive misuse	1.25 (1.25 - 1.26)	1.3 (1.13 - 1.49)
Hypertension - Stroke & TIA	1.25 (1.25 - 1.25)	1.29 (1.19 - 1.4)
Chronic kidney disease - Glaucoma	1.25 (1.23 - 1.27)	1.26 (1.02 - 1.55)
Depression - Blindness & low vision	1.24 (1.22 - 1.26)	1.27 (1.05 - 1.55)
Treated dyspepsia - COPD	1.24 (1.21 - 1.26)	NS
Painful condition - Chronic kidney disease	1.23 (1.22 - 1.24)	1.29 (1.1 - 1.46)
Asthma (currently treated) - Coronary heart disease	1.23 (1.2 - 1.25)	1.22 (1.01 - 1.55)
Hearing loss - Heart failure	1.23 (1.22 - 1.23)	1.28 (1.11 - 1.45)
Coronary heart disease - Thyroid disorders	1.22 (1.22 - 1.23)	1.25 (1.12 - 1.42)
Diverticular disease - Atrial fibrillation (AF)	1.22 (1.21 - 1.24)	1.25 (1.08 - 1.47)
Hearing loss - Any cancer last 5 years	1.22 (1.21 - 1.23)	1.25 (1.08 - 1.43)
Thyroid disorders - Diverticular disease	1.22 (1.21 - 1.23)	1.19 (1.08 - 1.44)

	RR (CI 99%)	ABC (CI 99%)
Thyroid disorders - Other psychoactive misuse	1.22 (1.21 - 1.23)	1.23 (1.09 - 1.44)
Stroke & TIA - Chronic kidney disease	1.21 (1.21 - 1.22)	1.25 (1.11 - 1.44)
Hearing loss - Blindness & low vision	1.21 (1.2 - 1.23)	1.26 (1.04 - 1.46)
Anxiety related disorders - Blindness & low vision	1.21 (1.19 - 1.23)	1.23 (1.01 - 1.52)
Depression - Stroke & TIA	1.21 (1.2 - 1.22)	1.25 (1.11 - 1.43)
Thyroid disorders - Chronic kidney disease	1.21 (1.2 - 1.22)	1.21 (1.08 - 1.43)
COPD - Anxiety related disorders	1.21 (1.19 - 1.23)	1.3 (1.04 - 1.5)
Hypertension - Rheumatoid arthritis	1.2 (1.2 - 1.21)	1.25 (1.13 - 1.37)
Painful condition - COPD	1.2 (1.18 - 1.22)	1.24 (1.0 - 1.49)
Painful condition - Thyroid disorders	1.19 (1.18 - 1.2)	1.23 (1.07 - 1.4)
Coronary heart disease - Blindness & low vision	1.19 (1.18 - 1.2)	1.23 (1.06 - 1.41)
Thyroid disorders - Anxiety related disorders	1.19 (1.18 - 1.2)	1.19 (1.07 - 1.41)
Treated constipation - Stroke & TIA	1.19 (1.18 - 1.19)	1.22 (1.11 - 1.36)
Rheumatoid arthritis - Anxiety related disorders	1.19 (1.17 - 1.2)	1.24 (1.04 - 1.45)
Anxiety related disorders - Dementia	1.18 (1.17 - 1.19)	1.15 (1.04 - 1.4)
Anxiety related disorders - Chronic kidney disease	1.18 (1.17 - 1.19)	1.23 (1.06 - 1.4)
Treated dyspepsia - Chronic kidney disease	1.18 (1.16 - 1.19)	1.22 (1.04 - 1.4)
Coronary heart disease - Treated constipation	1.17 (1.17 - 1.17)	1.22 (1.11 - 1.33)
Hypertension - Atrial fibrillation (AF)	1.17 (1.17 - 1.17)	1.22 (1.11 - 1.31)
Thyroid disorders - Atrial fibrillation (AF)	1.17 (1.16 - 1.18)	1.16 (1.04 - 1.4)
Hearing loss - Glaucoma	1.17 (1.15 - 1.18)	NS
Rheumatoid arthritis - Stroke & TIA	1.17 (1.16 - 1.18)	1.25 (1.02 - 1.4)
Chronic kidney disease - Diverticular disease	1.17 (1.15 - 1.18)	1.19 (1.04 - 1.42)
Stroke & TIA - Dementia	1.16 (1.16 - 1.17)	1.19 (1.03 - 1.35)
Painful condition - Coronary heart disease	1.16 (1.15 - 1.16)	1.24 (1.06 - 1.36)
Hearing loss - Atrial fibrillation (AF)	1.15 (1.14 - 1.16)	1.18 (1.04 - 1.35)
Thyroid disorders - Stroke & TIA	1.15 (1.14 - 1.16)	1.24 (1.03 - 1.35)
Hearing loss - Stroke & TIA	1.15 (1.14 - 1.15)	1.19 (1.04 - 1.33)
Hypertension - Heart failure	1.14 (1.14 - 1.15)	1.2 (1.08 - 1.31)
Hypertension - Coronary heart disease	1.13 (1.13 - 1.13)	1.19 (1.09 - 1.25)
Coronary heart disease - Hearing loss	1.12 (1.12 - 1.12)	1.16 (1.05 - 1.28)
Hearing loss - Chronic kidney disease	1.11 (1.11 - 1.12)	1.15 (1.03 - 1.32)
Thyroid disorders - Treated constipation	1.11 (1.11 - 1.12)	1.14 (1.01 - 1.3)
Hypertension - Other psychoactive misuse	1.11 (1.11 - 1.12)	1.16 (1.06 - 1.25)
Hearing loss - Treated constipation	1.11 (1.11 - 1.11)	1.13 (1.03 - 1.27)
Coronary heart disease - Anxiety related disorders	1.1 (1.1 - 1.11)	1.16 (1.02 - 1.29)
Hypertension - Any cancer last 5 years	1.09 (1.08 - 1.09)	NS
Hypertension - Diverticular disease	1.08 (1.08 - 1.08)	1.1 (1.01 - 1.23)
Hypertension - Thyroid disorders	1.07 (1.06 - 1.07)	NS
Other psychoactive misuse - Diverticular disease	NS	1.19 (1.01 - 1.40)
Treated constipation - Chronic kidney disease	NS	1.17 (1.01 - 1.27)
Hypertension - Depression	0.939 (0.936 - 0.942)	NS
Hypertension - COPD	0.904 (0.898 - 0.91)	NS
Coronary heart disease - Dementia	0.835 (0.83 - 0.84)	0.844 (0.752 - 0.966)
Hypertension - Dementia	0.832 (0.829 - 0.835)	0.83 (0.754 - 0.912)
Treated dyspepsia - Atrial fibrillation (AF)	0.825 (0.814 - 0.837)	NS
Chronic kidney disease - Dementia	0.82 (0.811 - 0.829)	0.825 (0.72 - 0.998)
Heart failure - Dementia	0.802 (0.792 - 0.813)	0.853 (0.699 - 0.989)
Hypertension - Parkinson's disease	0.764 (0.729 - 0.801)	NS
Coronary heart disease - Treated dyspepsia	0.752 (0.745 - 0.758)	0.753 (0.668 - 0.896)
Painful condition - Dementia	0.695 (0.686 - 0.704)	0.694 (0.587 - 0.86)
Treated dyspepsia - Stroke & TIA	0.673 (0.663 - 0.683)	0.708 (0.575 - 0.834)
Asthma (currently treated) - Dementia	0.505 (0.469 - 0.543)	0.58 (0.408 - 0.847)
Rheumatoid arthritis - Parkinson's disease	0.394 (0.273 - 0.57)	NS
Thyroid disorders - Prostate disorders	0.351 (0.305 - 0.405)	0.491 (0.313 - 0.771)

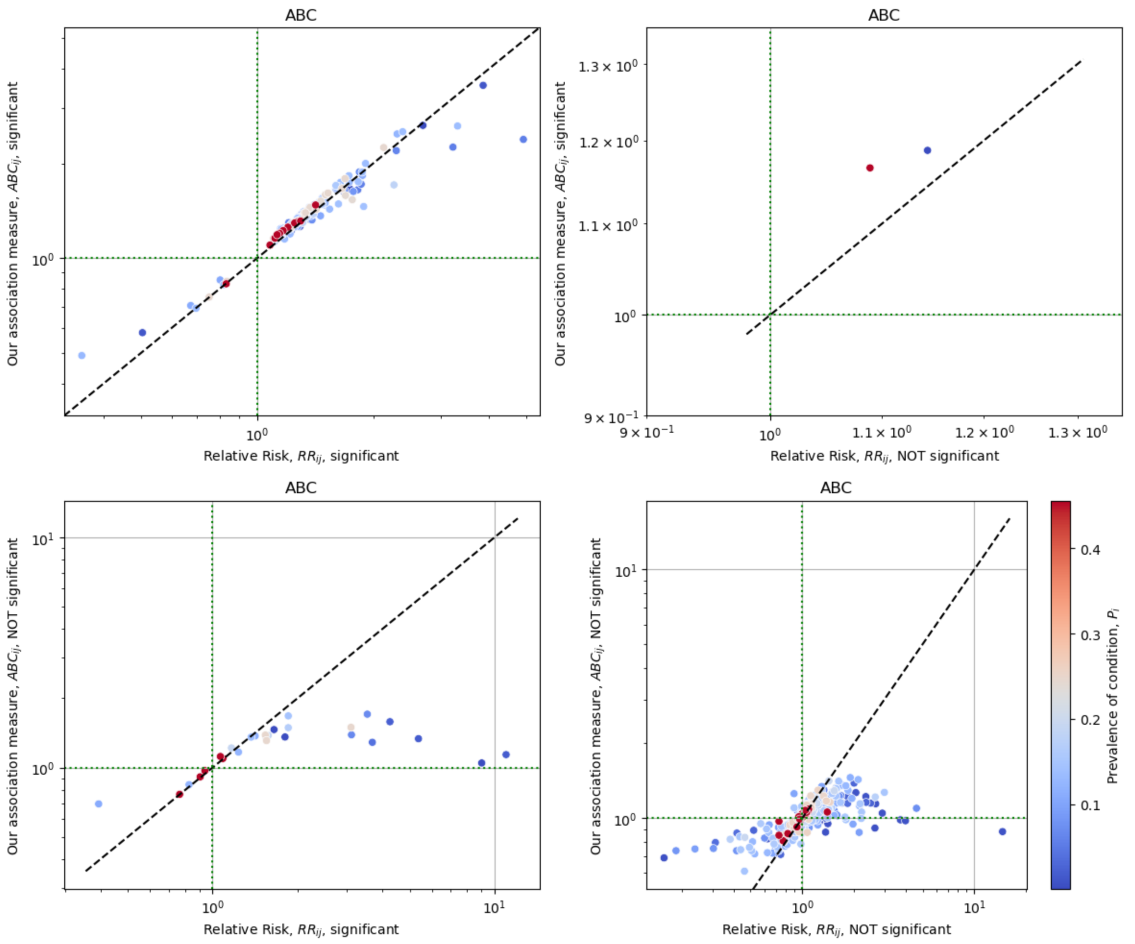

Figure 4: **Relation between RR and ABC associations for the 780 associations included in our study**, with each point representing a single association whose colour reflects the highest prevalence of the two LTCs. From left to right, top to bottom: (A) associations found significant by both measures (165 pairs), (B) associations found significant by ABC but not by RR (2 pairs), (C) associations found significant by RR but not by ABC (27 pairs), and (D) associations not found significant by either measure (547 pairs).

Table 3: **Associations for which significant differences between men and women are found by ABC ($p < 0.05$).** Plots of the distributions are also shown in Figure S5.

ABC (significant)	Men	Women	p-value
Depression - Anxiety & related disorders	3.35 (2.57 - 4.42)	2.21 (2.0 - 2.47)	<0.001
Depression - Painful condition	2.02 (1.44 - 2.75)	1.54 (1.35 - 1.76)	0.027
Anxiety & related disorders - Treated constipation	2.07 (1.63 - 2.57)	1.63 (1.46 - 1.79)	0.008
Depression - Dementia	2.15 (1.61 - 2.95)	1.77 (1.57 - 1.99)	0.043
Other psychoactive misuse - Dementia	1.72 (1.26 - 2.36)	1.38 (1.19 - 1.58)	0.044
Thyroid disorders - Chronic kidney disease (CKD)	1.51 (1.08 - 2.15)	1.17 (1.01 - 1.39)	0.038
Chronic kidney disease (CKD) - Heart failure	2.16 (1.77 - 2.75)	1.87 (1.61 - 2.16)	0.047
Painful condition - Treated constipation	1.86 (1.49 - 2.35)	1.58 (1.43 - 1.77)	0.043
Depression - Stroke & TIA	1.46 (1.12 - 1.94)	1.21 (1.04 - 1.4)	0.046
Hypertension - Chronic kidney disease (CKD)	1.62 (1.39 - 1.89)	1.45 (1.32 - 1.57)	0.046
CHD - Other psychoactive misuse	1.6 (1.31 - 1.94)	1.86 (1.67 - 2.04)	0.049
Diabetes - Chronic kidney disease (CKD)	1.52 (1.16 - 2.06)	1.97 (1.66 - 2.32)	0.029

Table 4: **Associations for which significant differences between men and women are found by RR ($p < 0.05$).** On the right–most column, indication whether these differences were also found to be significant by ABC ($p < 0.05$).

RR (significant)	Men	Women	also ABC
Depression - Anxiety related disorders	3.64 (3.55 - 3.73)	2.14 (2.13 - 2.14)	Yes
Depression - Irritable bowel syndrome	2.49 (2.01 - 3.08)	1.65 (1.59 - 1.71)	No
Anxiety related disorders - Irritable bowel syndrome	2.29 (1.77 - 2.96)	1.59 (1.53 - 1.65)	No
Depression - Painful condition	2.14 (2.06 - 2.22)	1.5 (1.49 - 1.51)	Yes
Depression - Dementia	2.36 (2.28 - 2.44)	1.73 (1.72 - 1.74)	Yes
Anxiety related disorders - Treated constipation	2.07 (2.03 - 2.11)	1.55 (1.55 - 1.56)	Yes
Treated constipation - Parkinson's disease	2.07 (1.88 - 2.27)	1.56 (1.46 - 1.66)	No
Other psychoactive misuse - Dementia	1.83 (1.76 - 1.9)	1.34 (1.33 - 1.35)	Yes
Thyroid disorders - Chronic kidney disease	1.62 (1.54 - 1.71)	1.15 (1.14 - 1.16)	Yes
Thyroid disorders - Rheumatoid arthritis	1.62 (1.5 - 1.74)	1.2 (1.19 - 1.21)	No
Chronic kidney disease - Heart failure	2.2 (2.16 - 2.24)	1.8 (1.79 - 1.82)	Yes
Painful condition - Treated constipation	1.89 (1.85 - 1.92)	1.54 (1.53 - 1.54)	Yes
Anxiety related disorders - Diverticular disease	1.69 (1.61 - 1.79)	1.35 (1.34 - 1.36)	No
Peripheral vascular disease - Heart failure	1.87 (1.78 - 1.97)	1.54 (1.48 - 1.6)	No
Depression - Treated constipation	1.98 (1.94 - 2.02)	1.66 (1.65 - 1.66)	No
Depression - Stroke & TIA	1.49 (1.44 - 1.53)	1.17 (1.16 - 1.18)	Yes
Painful condition - Rheumatoid arthritis	1.73 (1.65 - 1.81)	1.42 (1.41 - 1.44)	No
Depression - Other psychoactive misuse	1.63 (1.56 - 1.71)	1.33 (1.32 - 1.34)	No
Rheumatoid arthritis - Peripheral vascular disease	1.7 (1.58 - 1.84)	1.41 (1.35 - 1.47)	No
Coronary heart disease - Thyroid disorders	1.49 (1.45 - 1.53)	1.22 (1.22 - 1.23)	No
Treated constipation - Diverticular disease	1.45 (1.42 - 1.49)	1.2 (1.2 - 1.21)	No
Chronic kidney disease - Atrial fibrillation (AF)	1.65 (1.61 - 1.68)	1.41 (1.4 - 1.43)	No
Anxiety related disorders - Other psychoactive misuse	1.45 (1.37 - 1.53)	1.22 (1.21 - 1.23)	No
Depression - Treated dyspepsia	1.66 (1.58 - 1.75)	1.44 (1.43 - 1.45)	No
Other psychoactive misuse - Treated constipation	1.45 (1.42 - 1.48)	1.26 (1.26 - 1.27)	No
Rheumatoid arthritis - Atrial fibrillation (AF)	1.41 (1.36 - 1.46)	1.22 (1.21 - 1.24)	No
Hypertension - Chronic kidney disease	1.56 (1.55 - 1.57)	1.38 (1.38 - 1.38)	Yes
Rheumatoid arthritis - Chronic kidney disease	1.51 (1.46 - 1.56)	1.33 (1.32 - 1.35)	No
Hypertension - Heart failure	1.28 (1.26 - 1.29)	1.11 (1.11 - 1.12)	No
Any cancer last 5 years - Diverticular disease	1.4 (1.34 - 1.46)	1.23 (1.21 - 1.26)	No
Diverticular disease - Atrial fibrillation (AF)	1.35 (1.29 - 1.4)	1.2 (1.18 - 1.21)	No
Hearing loss - Heart failure	1.32 (1.29 - 1.35)	1.18 (1.17 - 1.19)	No
Coronary heart disease - Rheumatoid arthritis	1.43 (1.4 - 1.46)	1.3 (1.29 - 1.31)	No
Hypertension - Other psychoactive misuse	1.2 (1.18 - 1.22)	1.08 (1.08 - 1.08)	No
Other psychoactive misuse - Chronic kidney disease	1.36 (1.31 - 1.41)	1.25 (1.24 - 1.26)	No
Hypertension - Atrial fibrillation (AF)	1.26 (1.24 - 1.27)	1.15 (1.14 - 1.15)	No
Hypertension - Rheumatoid arthritis	1.28 (1.26 - 1.3)	1.18 (1.17 - 1.18)	No
Hypertension - Stroke & TIA	1.33 (1.32 - 1.34)	1.24 (1.23 - 1.24)	No
Coronary heart disease - Chronic kidney disease	1.59 (1.57 - 1.61)	1.5 (1.5 - 1.51)	No
Rheumatoid arthritis - Heart failure	1.7 (1.64 - 1.76)	1.61 (1.59 - 1.63)	No
Other psychoactive misuse - Atrial fibrillation	1.44 (1.39 - 1.49)	1.36 (1.35 - 1.37)	No
Other psychoactive misuse - Stroke & TIA	1.47 (1.43 - 1.51)	1.39 (1.38 - 1.4)	No
Hypertension - Diabetes	1.33 (1.31 - 1.36)	1.26 (1.26 - 1.27)	No
Stroke & TIA - Diverticular disease	1.32 (1.28 - 1.37)	1.27 (1.26 - 1.28)	No
Hypertension - Coronary heart disease	1.16 (1.16 - 1.17)	1.13 (1.13 - 1.13)	No
Coronary heart disease - Treated constipation	1.19 (1.18 - 1.2)	1.17 (1.17 - 1.18)	No
Stroke & TIA - Atrial fibrillation (AF)	1.56 (1.54 - 1.59)	1.61 (1.6 - 1.62)	No
Atrial fibrillation (AF) - Heart failure	2.26 (2.22 - 2.3)	2.32 (2.3 - 2.33)	No
Stroke & TIA - Heart failure	1.27 (1.23 - 1.3)	1.34 (1.32 - 1.35)	No
Coronary heart disease - Atrial fibrillation (AF)	1.45 (1.43 - 1.46)	1.52 (1.51 - 1.53)	No
Coronary heart disease - Dementia	0.78 (0.76 - 0.81)	0.87 (0.86 - 0.87)	No
Hearing loss - Diverticular disease	1.29 (1.25 - 1.32)	1.37 (1.36 - 1.38)	No
Coronary heart disease - Stroke & TIA	1.26 (1.25 - 1.27)	1.36 (1.35 - 1.37)	No
COPD - Heart failure	1.77 (1.72 - 1.83)	1.89 (1.85 - 1.93)	No
Treated constipation - Dementia	1.34 (1.31 - 1.38)	1.46 (1.45 - 1.46)	No

RR (significant)	Men	Women	also ABC
Hearing loss - COPD	1.26 (1.23 - 1.3)	1.4 (1.38 - 1.42)	No
Coronary heart disease - Treated dyspepsia	0.63 (0.60 - 0.66)	0.8 (0.79 - 0.81)	No
Coronary heart disease - Heart failure	2.0 (1.98 - 2.02)	2.17 (2.17 - 2.18)	No
Diabetes - Heart failure	1.36 (1.31 - 1.42)	1.54 (1.52 - 1.57)	No
Coronary heart disease - Other psychoactive misuse	1.57 (1.54 - 1.59)	1.76 (1.75 - 1.76)	Yes
Diabetes - Other psychoactive misuse	1.46 (1.4 - 1.54)	1.66 (1.65 - 1.68)	No
Diabetes - Chronic kidney disease	1.58 (1.53 - 1.64)	1.94 (1.92 - 1.96)	Yes

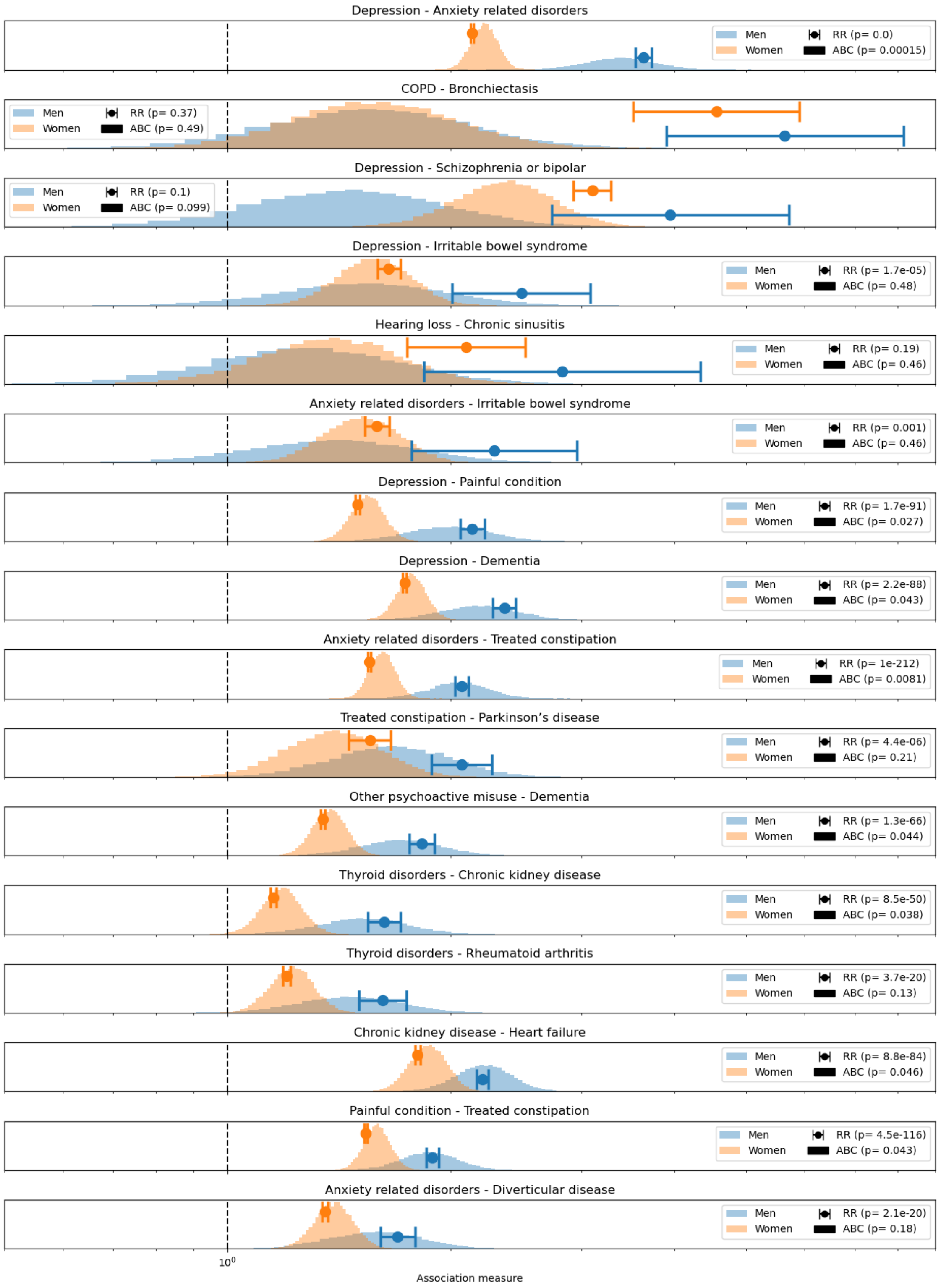

Figure 5: **Comparison of differences between associations by sex using ABC (shaded distributions) and RR methods (dots with error bars).** In the legend, the p-values of the overlap of distributions for each method.

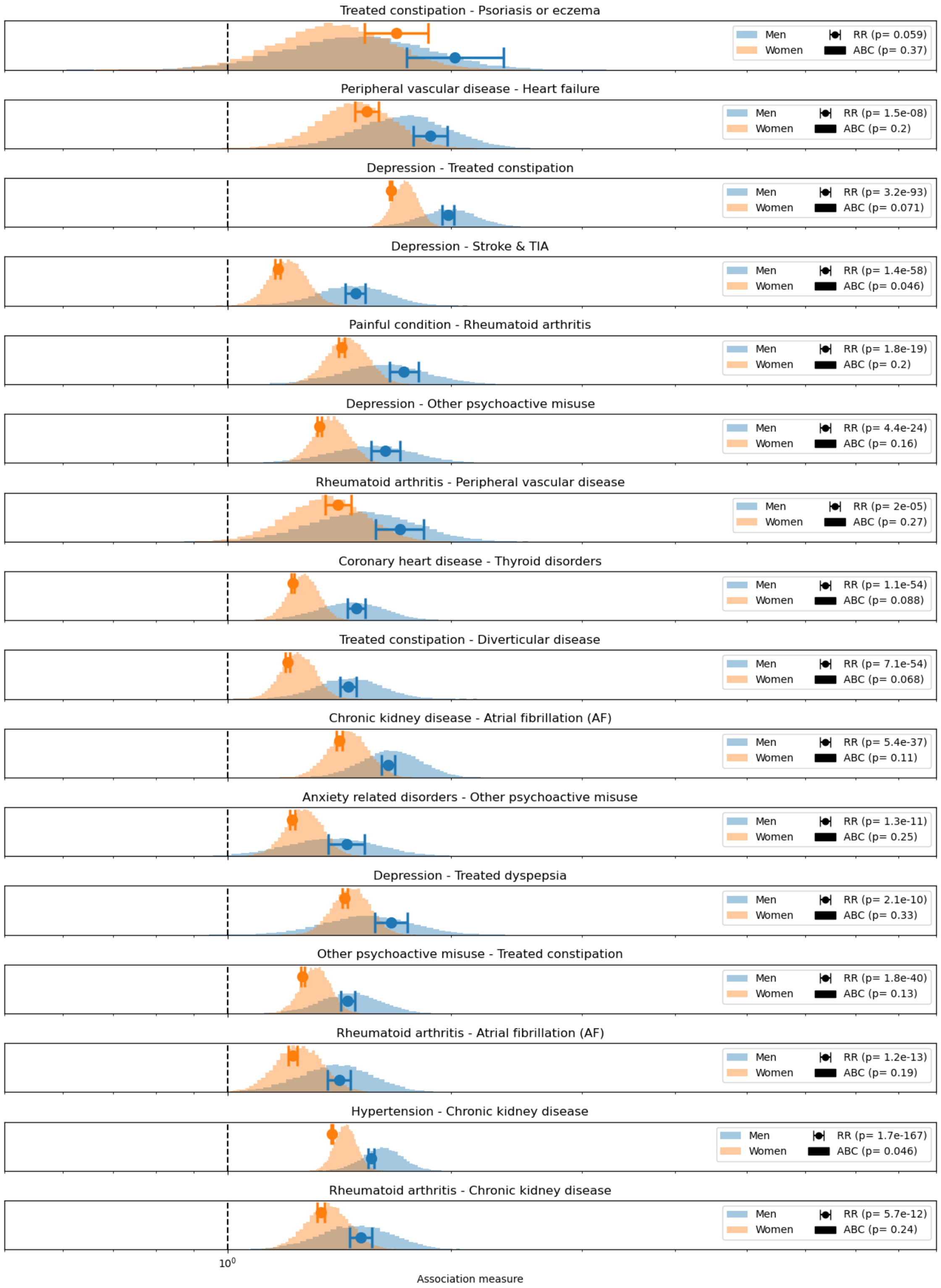

Figure 6: **Comparison of differences between associations by sex using ABC (shaded distributions) and RR methods (dots with error bars).** In the legend, the p-values of the overlap of distributions for each method.

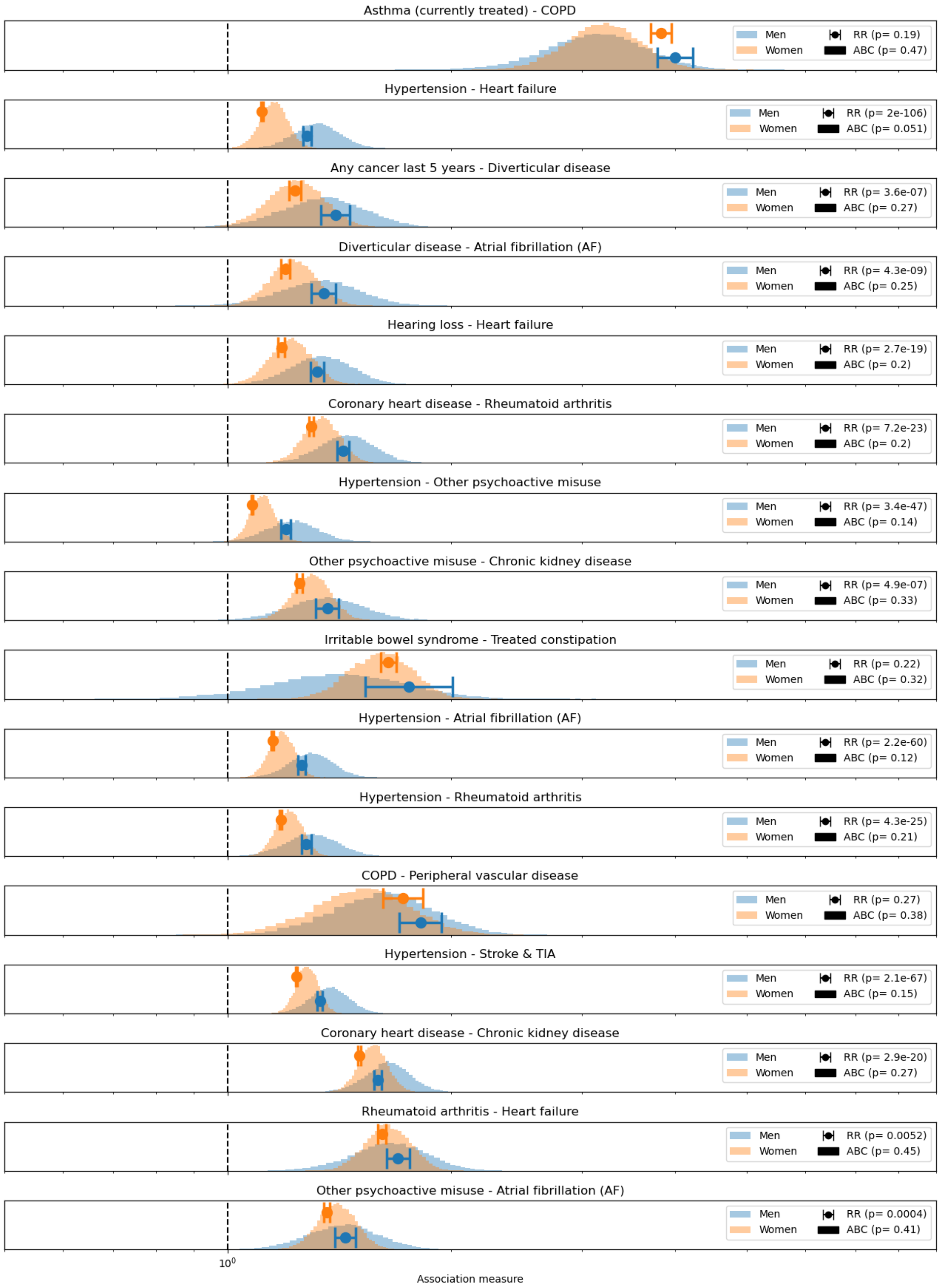

Figure 7: **Comparison of differences between associations by sex using ABC (shaded distributions) and RR methods (dots with error bars).** In the legend, the p-values of the overlap of distributions for each method.

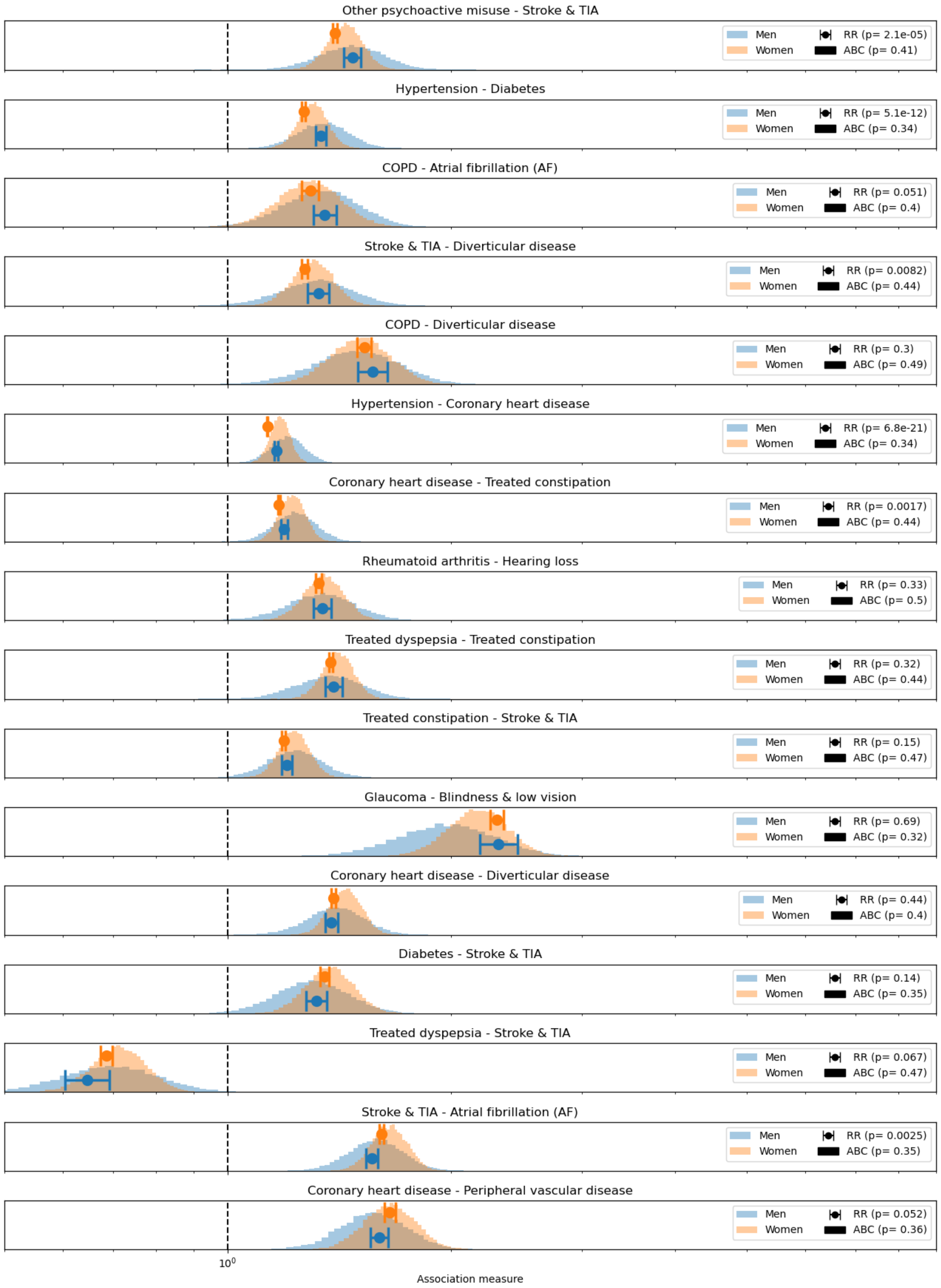

Figure 8: **Comparison of differences between associations by sex using ABC (shaded distributions) and RR methods (dots with error bars).** In the legend, the p-values of the overlap of distributions for each method.

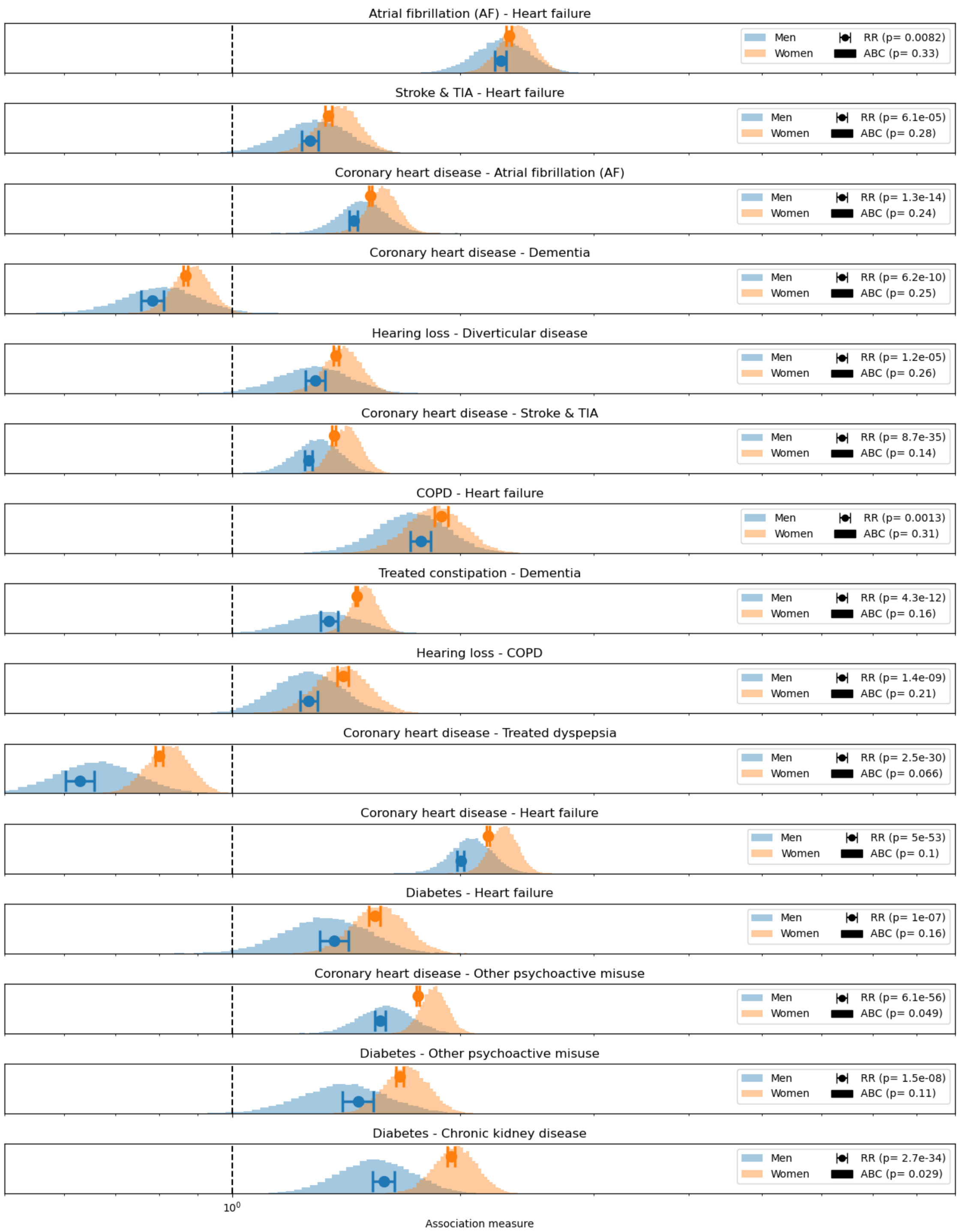

Figure 9: **Comparison of differences between associations by sex using ABC (shaded distributions) and RR methods (dots with error bars).** In the legend, the p-values of the overlap of distributions for each method.

ABC association network	RR association network
Asthma (currently treated)	
COPD	
Bronchiectasis	
Alcohol problems	
	Asthma (currently treated)
	COPD
	Bronchiectasis
	Alcohol problems
Hypertension	Hypertension
Coronary heart disease	Coronary heart disease
Diabetes	Diabetes
Rheumatoid arthritis	Rheumatoid arthritis
Chronic kidney disease	Chronic kidney disease
Atrial fibrillation (AF)	Atrial fibrillation (AF)
Heart failure	Heart failure
Peripheral vascular disease	
Thyroid disorders	
Other psychoactive misuse	
Stroke & TIA	
	Thyroid disorders
	Other psychoactive misuse
	Stroke & TIA
Depression	Depression
Painful condition	Painful condition
Parkinson's disease	Parkinson's disease
Treated dyspepsia	Treated dyspepsia
Anxiety related disorders	Anxiety related disorders
Treated constipation	Treated constipation
Dementia	Dementia
Irritable bowel syndrome	
Schizophrenia or bipolar	
	Multiple sclerosis
	Epilepsy (currently treated)
Psoriasis or eczema	
Diverticular disease	
	Psoriasis or eczema
	Diverticular disease
Prostate disorders	Prostate disorders
Glaucoma	Glaucoma
Hearing loss	Hearing Loss
Any cancer last 5 years	Any cancer last 5 years
Blindness & low vision	Blindness & low vision
Chronic sinusitis	
	Chronic sinusitis
	Irritable bowel syndrome
	Migraine
	Anorexia or bulimia
	Schizophrenia or bipolar
	Peripheral vascular disease

Table 5: **Clusters of conditions found in ABC and RR networks via the Clauset–Newman–Moore greedy modularity maximisation algorithm [15].**

Table 6: **Average associations in our oldest–old cohort found by our ABC approach and by relative risk (RR).**

	ABC (CI 99%)	RR
Chronic obstructive pulmonary disease (COPD)	1.34 (1.25 - 1.44)	1.44
Heart failure	1.31 (1.24 - 1.38)	1.23
Depression	1.29 (1.23 - 1.37)	1.23
Irritable bowel syndrome (IBS)	1.27 (1.18 - 1.39)	1.52
Diverticular disease of intestine	1.26 (1.19 - 1.34)	1.2
Other psychoactive misuse	1.26 (1.19 - 1.34)	1.14
Treated constipation	1.25 (1.19 - 1.32)	1.25
Coronary heart disease (CHD)	1.23 (1.17 - 1.29)	1.17
Peripheral vascular disease	1.23 (1.14 - 1.33)	1.19
Anxiety & other neurotic, stress related & somatoform disorders	1.22 (1.16 - 1.3)	1.18
Blindness & low vision	1.21 (1.13 - 1.3)	1.17
Chronic kidney disease (CKD)	1.21 (1.15 - 1.28)	1.15
Rheumatoid arthritis & related disorders	1.21 (1.13 - 1.29)	1.1
Atrial fibrillation (AF)	1.2 (1.13 - 1.28)	1.14
Asthma (currently treated)	1.19 (1.1 - 1.28)	1.1
Stroke & transient ischaemic attack (TIA)	1.18 (1.12 - 1.26)	1.12
Prostate disorders	1.18 (1.08 - 1.27)	1.18
Hearing loss	1.18 (1.11 - 1.24)	1.14
Diabetes	1.16 (1.09 - 1.24)	1.12
Schizophrenia (and related non-organic psychosis) or bipolar disorder	1.14 (1.04 - 1.27)	1.32
Alcohol problems	1.14 (1.03 - 1.26)	1.09
Psoriasis or eczema	1.14 (1.03 - 1.26)	1.12
Thyroid disorders	1.13 (1.07 - 1.21)	1.06
Painful condition	1.13 (1.07 - 1.2)	1.11
Treated dyspepsia	1.12 (1.05 - 1.19)	1.07
Chronic sinusitis	1.11 (0.992 - 1.24)	1.21
New diagnosis of cancer in last five years	1.1 (1.03 - 1.18)	1.03
Parkinson's disease	1.1 (0.996 - 1.21)	1.03
Glaucoma	1.09 (1.01 - 1.17)	1.06
Bronchiectasis	1.07 (0.948 - 1.21)	1.1
Dementia	1.07 (1.01 - 1.14)	1.07
Anorexia or Bulimia	1.07 (0.951 - 1.19)	1.33
Epilepsy (currently treated)	1.06 (0.954 - 1.18)	1.04
Inflammatory bowel disease	1.06 (0.949 - 1.18)	1.0
Hypertension	1.05 (1.0 - 1.1)	1.04
Migraine	1.05 (0.922 - 1.19)	1.26
Multiple sclerosis	1.03 (0.91 - 1.16)	1.05
Chronic liver disease	1.01 (0.887 - 1.15)	1.0
Learning disability	0.988 (0.868 - 1.12)	1.0

2.1 Results stratified by sex

Out of the 780 possible associations between the 40 LTCs, the ABC measure found 68 (8.7%) significant associations in men and 125 (16.0%) in women, while RR found 113 (14.5%) significant associations in men and 155 (19.9%) in women (Figure 10).

The top ten associations found by RR in either sex, along with their ABC values, are listed in Tables 7 and 8. Out of the associations found by RR, only three are also found to be significant by ABC in men (active asthma with COPD, depression with anxiety disorders, and COPD with alcohol problems) and only two in women (asthma with COPD and depression with schizophrenia or bipolar disorders). The top association found by RR in men is between peripheral vascular disease and anorexia or bulimia (6.85 [99% CI 2.9 - 16.2]), which was not found to be significant by ABC. The top three associations found by RR correspond to alcohol problems with learning disability, irritable bowel syndrome with migraine and schizophrenia or bipolar disorders with anorexia or bulimia, with values 55.4 (99% CI 15.2 - 202), 12.0 (5.08 - 28.4), and 9.94 (4.21 - 23.5) times what expected by chance, respectively, none of which were found to be significant by ABC.

Regarding negative associations —implying mutual exclusiveness between the two LTCs— our ABC measure only found one significant association in men and six in women, while RR found

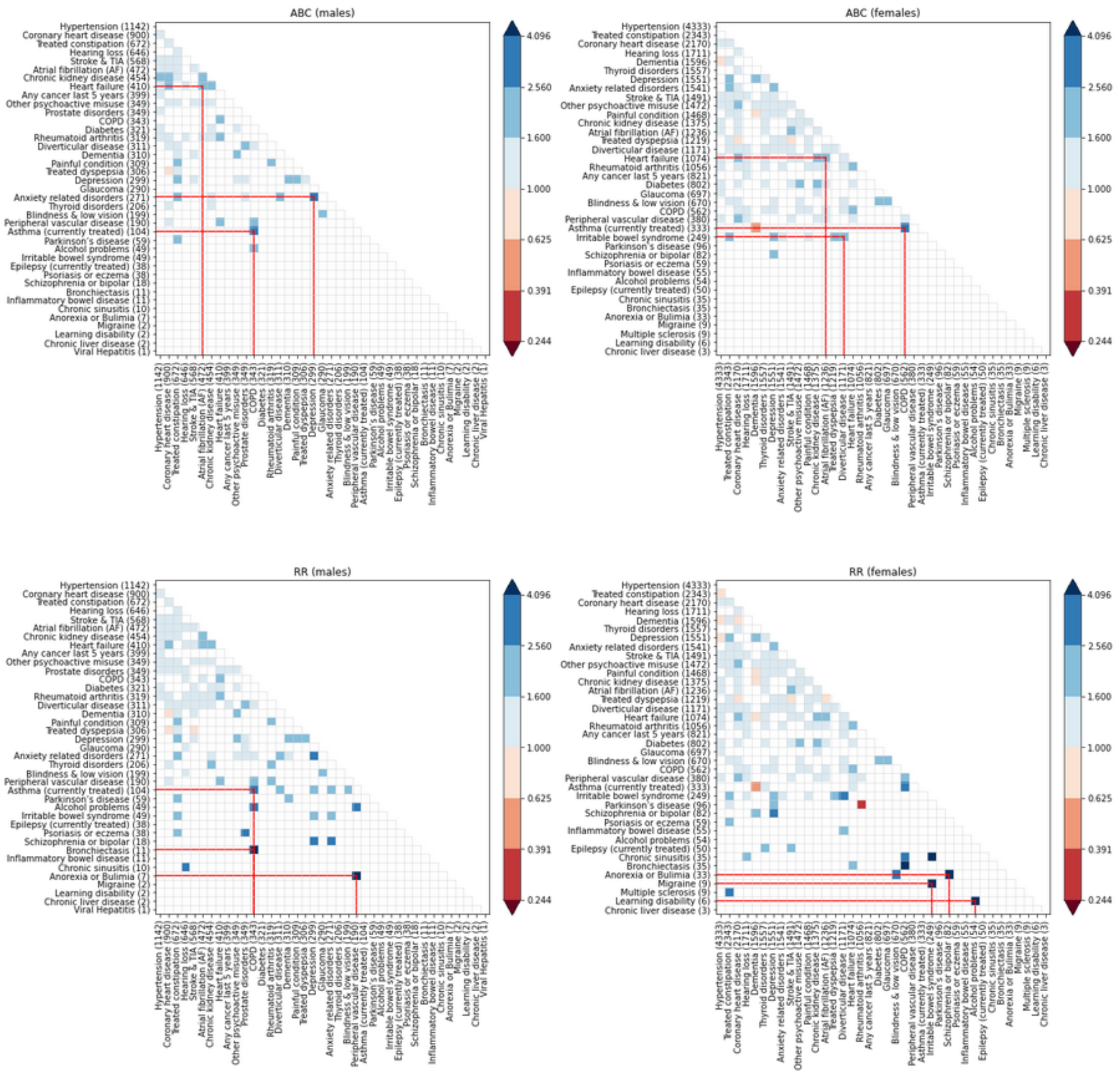

Figure 10: **Associations Beyond Chance (top) and relative risk (bottom) between the studied 40 LTCs in men (left) and women (right) in the oldest–old cohort.** Blue cells correspond to positive associations (ABC, RR>1), red cells correspond to negative associations (ABC, RR<1), and white cells correspond to associations not deemed significant. The top three positive associations for each method are highlighted. Note that LTCs are sorted by their prevalence within the cohort, with counts in brackets. Association values are coloured using a logarithmic scale.

	RR (CI 99%)	ABC (CI 99%)
Peripheral vascular disease - Anorexia or Bulimia	6.85 (2.9 - 16.2)	NS
**COPD - Bronchiectasis**	5.64 (3.9 - 8.14)	NS
**Asthma (currently treated) - COPD**	4.0 (3.79 - 4.23)	3.15 (2.12 - 4.65)
**Depression - Schizophrenia or bipolar disorder**	3.95 (2.74 - 5.71)	NS
Anxiety & related disorders - Schizophrenia or bipolar disorder	3.74 (2.43 - 5.74)	NS
Depression - Anxiety & related disorders	3.64 (3.55 - 3.73)	3.35 (2.57 - 4.42)
COPD - Alcohol problems	3.25 (2.82 - 3.75)	2.04 (1.13 - 3.63)
Hearing loss - Chronic sinusitis	2.82 (1.84 - 4.33)	NS
Prostate disorders - Psoriasis or eczema	2.75 (2.22 - 3.41)	NS
Alcohol problems - Peripheral vascular disease	2.61 (1.89 - 3.6)	NS

Table 7: **Top ten positive associations in men ($>$ 90yo) found by relative risk (RR), along the value given by our approach (ABC) to these.** ‘NS’ marks an association not deemed to be significant by the method. In bold, associations that are in the top ten in both sexes.

three in men and 12 in women (Tables 9 and 10). All negative associations found by using ABC were also found by using RR, and all associations found in men by either method were also found

	RR (CI 99%)	ABC (CI 99%)
Alcohol problems - Learning disability	55.4 (15.2 - 202)	NS
Irritable bowel syndrome (IBS) - Migraine	12.0 (5.08 - 28.4)	NS
Schizophrenia or bipolar disorder - Anorexia or bulimia	9.94 (4.21 - 23.5)	NS
Irritable bowel syndrome (IBS) - Chronic sinusitis	5.15 (3.07 - 8.61)	NS
**COPD - Bronchiectasis**	4.56 (3.53 - 5.9)	NS
**Asthma (currently treated) - COPD**	3.83 (3.71 - 3.96)	3.21 (2.41 - 4.33)
COPD - Chronic Sinusitis	3.65 (2.64 - 5.03)	NS
Treated constipation - Multiple sclerosis	3.4 (2.47 - 4.69)	NS
**Depression - Schizophrenia or bipolar disorder**	3.1 (2.93 - 3.29)	2.41 (1.61 - 3.55)
Blindness & low vision - Anorexia or Bulimia	2.84 (1.97 - 4.1)	NS

Table 8: **Top ten positive associations in women (> 90yo) found by relative risk (RR), along the value given by our approach (ABC) to these.** 'NS' marks an association not deemed to be significant by the method. In bold, associations that are in the top ten in both sexes.

in women. Dementia appears in one negative association in men and six negative associations in women.

	RR (CI 99%)	ABC (CI 99%)
**Coronary heart disease - Treated dyspepsia**	0.629 (0.602 - 0.658)	0.673 (0.478 - 0.916)
**Treated dyspepsia - Stroke & TIA**	0.647 (0.604 - 0.693)	NS
**Coronary heart disease - Dementia**	0.784 (0.757 - 0.812)	NS

Table 9: **Negative associations found by RR and ABC association measures in men.** 'NS' marks that an association is not deemed to be significant due to lack of evidence. Pairs are sorted in ascending order by their RR association values. In bold, negative associations found by RR in both sexes.

	RR (CI 99%)	ABC (CI 99%)
Rheumatoid arthritis - Parkinsons's disease	0.265 (0.113 - 0.626)	NS
Asthma (currently treated) - Dementia	0.456 (0.414 - 0.501)	0.556 (0.382 - 0.835)
Painful condition - Dementia	0.662 (0.653 - 0.672)	0.683 (0.559 - 0.82)
**Treated dyspepsia - Stroke & TIA**	0.686 (0.674 - 0.699)	0.723 (0.574 - 0.883)
**Coronary heart disease - Treated dyspepsia**	0.8 (0.792 - 0.809)	0.821 (0.695 - 0.973)
Treated dyspepsia - Atrial fibrillation (AF)	0.804 (0.789 - 0.819)	NS
Hypertension - Dementia	0.807 (0.804 - 0.81)	0.795 (0.714 - 0.893)
Heart failure - Dementia	0.827 (0.814 - 0.84)	NS
Chronic kidney disease (CKD) - Dementia	0.834 (0.824 - 0.844)	NS
**Coronary heart disease - Dementia**	0.868 (0.861 - 0.874)	NS
Hypertension - Depression	0.901 (0.898 - 0.904)	NS
Hypertension - Treated constipation	0.943 (0.941 - 0.945)	NS

Table 10: **Negative associations found by RR and ABC association measures in women.** 'NS' marks that an association is not deemed to be significant due to lack of evidence. Pairs are sorted in ascending order by their RR association values. In bold, negative associations found by RR in both sexes.

	men	women	p-value
Depression - IBS	2.49 (2.01 - 3.08)	1.65 (1.59 - 1.71)	< 0.001
Anxiety & related disorders - IBS	2.29 (1.77 - 2.96)	1.59 (1.53 - 1.65)	0.001
Treated constipation - Parkinson's disease	2.07 (1.88 - 2.27)	1.56 (1.46 - 1.66)	< 0.001
Thyroid disorders - Rheumatoid arthritis	1.62 (1.5 - 1.74)	1.2 (1.19 - 1.21)	< 0.001
Anxiety & related disorders - Diverticular disease	1.69 (1.61 - 1.79)	1.35 (1.34 - 1.36)	< 0.001

Table 11: **Top five significant differences in RR values between sex for associations whose differences between sex are not deemed significant by our approach (p-value < 0.05).**

ABC network (men)	RR network (men)	ABC network (women)	RR network (women)
	Anorexia or bulimia		
	Bronchiectasis		
Asthma (currently treated)	Asthma (currently treated)	Asthma (currently treated)	
COPD	COPD	COPD	
Alcohol problems	Alcohol problems		Alcohol problems
	Peripheral vascular disease		
		Hearing loss	
		Any cancer last 5 years	
			Learning disability
			Asthma (currently treated)
			Bronchiectasis
			COPD
Peripheral vascular disease		Peripheral vascular disease	Peripheral vascular disease
Hypertension	Hypertension	Hypertension	Hypertension
Coronary heart disease	Coronary heart disease	Coronary heart disease	Coronary heart disease
Diabetes	Diabetes	Diabetes	Diabetes
Thyroid disorders	Thyroid disorders	Thyroid disorders	Thyroid disorders
Rheumatoid arthritis	Rheumatoid arthritis	Rheumatoid arthritis	Rheumatoid arthritis
Stroke & TIA	Stroke & TIA	Stroke & TIA	Stroke & TIA
Chronic kidney disease	Chronic kidney disease	Chronic kidney disease	Chronic kidney disease
Atrial fibrillation (AF)	Atrial fibrillation (AF)	Atrial fibrillation (AF)	Atrial fibrillation (AF)
Heart failure	Heart failure	Heart failure	Heart failure
Other psychoactive misuse		Other psychoactive misuse	Other psychoactive misuse
Hearing loss			
Any cancer last 5 years			
			Epilepsy (currently treated)
	Hearing loss		Hearing loss
	Any cancer last 5 years		Any cancer last 5 years
Glaucoma	Glaucoma	Glaucoma	
Blindness & low vision	Blindness & low vision	Blindness & low vision	
	Chronic sinusitis		Chronic sinusitis
	Diverticular disease		Diverticular disease
	Psoriasis or eczema		
			Irritable bowel syndrome
			Inflammatory bowel disease
			Migraine
	Prostate disorders		
Prostate disorders			
	Other psychoactive misuse		
Diverticular disease		Diverticular disease	
	Irritable bowel syndrome	Irritable bowel syndrome	
Depression	Depression	Depression	Depression
Painful condition	Painful condition	Painful condition	Painful condition
Treated dyspepsia	Treated dyspepsia	Treated dyspepsia	Treated dyspepsia
Anxiety related disorders	Anxiety related disorders	Anxiety related disorders	Anxiety related disorders
Treated constipation	Treated constipation	Treated constipation	Treated constipation
Dementia	Dementia	Dementia	Dementia
Parkinson's disease	Parkinson's disease		Parkinson's disease
	Schizophrenia or bipolar	Schizophrenia or bipolar	Schizophrenia or bipolar
			Anorexia or bulimia
			Multiple Sclerosis
			Glaucoma
			Blindness & low vision
			Stroke & TIA
			Psoriasis or eczema

Table 12: Clusters found in ABC and RR networks (stratified by sex) via the Clauset–Newman–Moore greedy modularity maximisation algorithm [15].

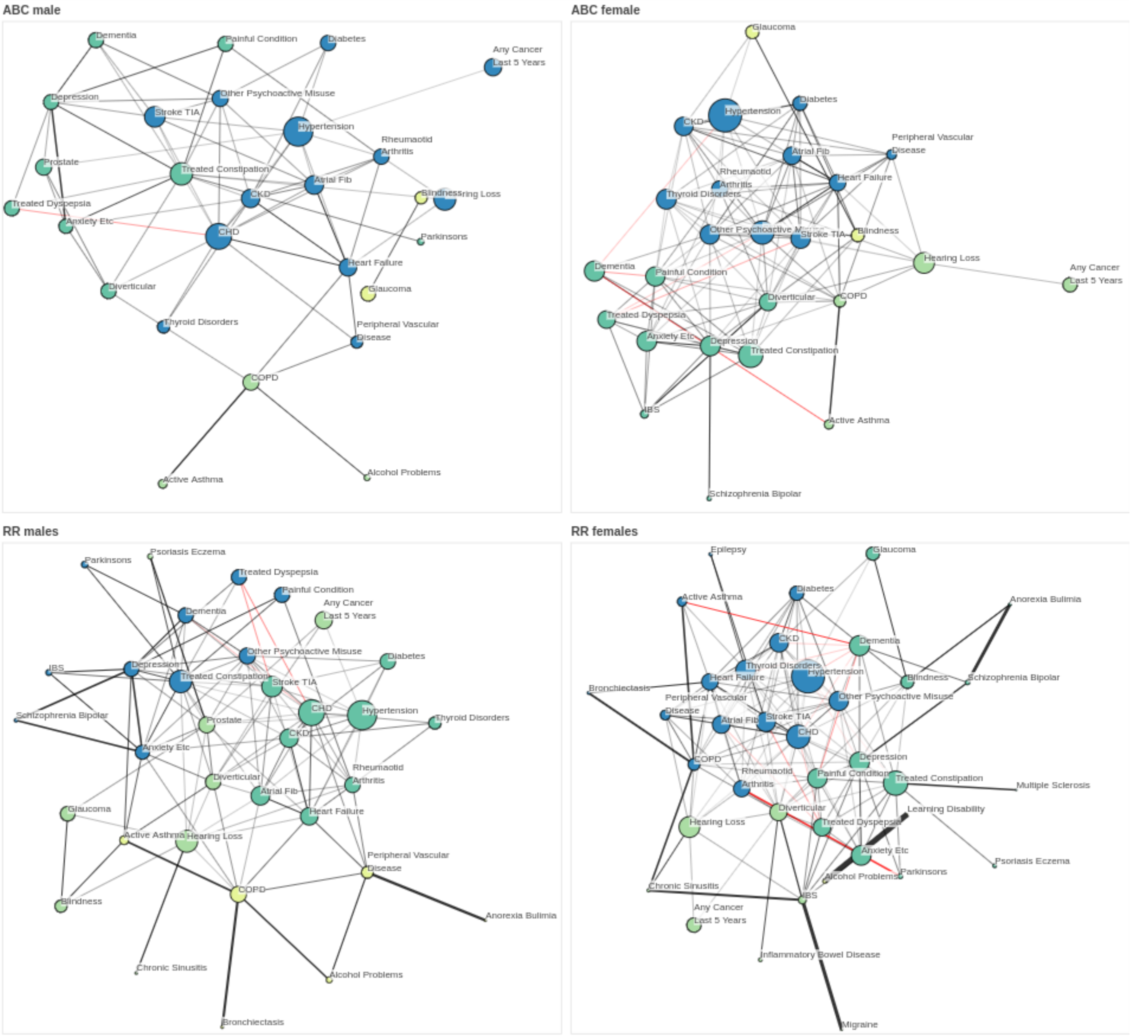

Figure 11: **Networks of associations with ABC (top) and RR (bottom) in men (left) and women (right).** Edge widths reflect the strength of the association, with positive associations in black and negative associations in red. In the ABC network, node size reflects the multimorbidity dependence score of the LTC.

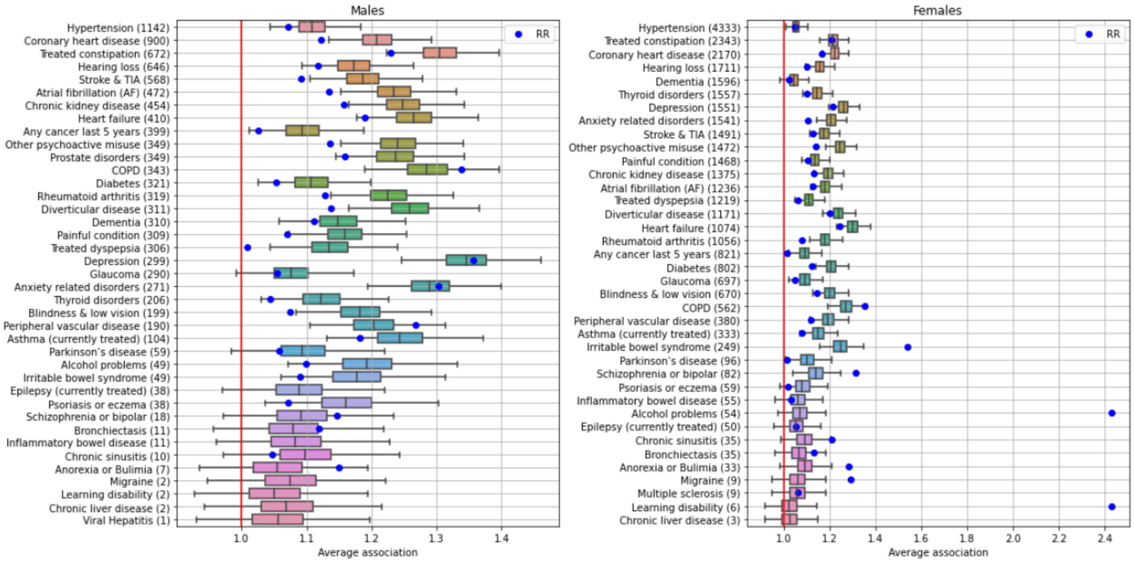

Figure 12: **Node strengths for the 40 LTCs in men (left) and women (right).** The red line corresponds to a score of zero, implying a strength equal to zero, i.e. no associations attached to the LTC. Boxes capture inter-quartiles of the distributions while whiskers extend to 99% confidence intervals. Blue circles denote node strengths as computed by relative risk.

	RR (90yo+)	ABC (90yo+)	RR (85yo+)	ABC (85yo+)
IBS - Migraine	11.0 (4.65 - 26.0)	NS	5.63 (4.23 - 7.5)	NS
Schizophrenia or bipolar - Anorexia or bulimia	9.01 (3.81 - 21.3)	NS	NS	NS
IBS - Chronic sinusitis	5.37 (3.5 - 8.25)	NS	3.37 (2.94 - 3.86)	1.72 (1.15 - 2.64)
COPD - Bronchiectasis	4.9 (4.22 - 5.71)	2.38 (1.24 - 4.24)	4.35 (4.19 - 4.52)	2.57 (1.99 - 3.67)
Prostate disorders - Psoriasis or eczema	4.26 (3.44 - 5.28)	NS	2.67 (2.48 - 2.87)	1.82 (1.28 - 2.62)
Asthma - COPD	3.86 (3.78 - 3.93)	3.54 (2.87 - 4.43)	3.46 (3.44 - 3.47)	2.88 (2.58 - 3.19)
Peripheral vascular disease - Anorexia or bulimia	3.69 (2.55 - 5.33)	NS	NS	NS
COPD - Chronic sinusitis	3.54 (2.86 - 4.39)	NS	2.24 (2.09 - 2.4)	1.66 (1.16 - 2.36)
Depression - Schizophrenia or bipolar	3.31 (3.15 - 3.48)	2.63 (1.89 - 3.84)	3.58 (3.54 - 3.63)	2.83 (2.36 - 3.29)
COPD - Alcohol problems	3.22 (2.91 - 3.57)	2.25 (1.28 - 3.4)	2.39 (2.33 - 2.46)	2.0 (1.59 - 2.51)

Table 13: **Top-ten RR associations in the 90 years old and above cohort, their corresponding ABC association values, and their corresponding RR and ABC association values for a wider cohort including patients 85 years old and above.** Parenthesis give the 99% confidence / credible intervals. Acronyms stand for IBS: Irritable Bowel Syndrome, SB: Schizophrenia (and related non-organic psychosis) or bipolar disorder, COPD: Chronic Obstructive Pulmonary Disease, P: Prostate disorders, PVD: Peripheral Vascular Disease.

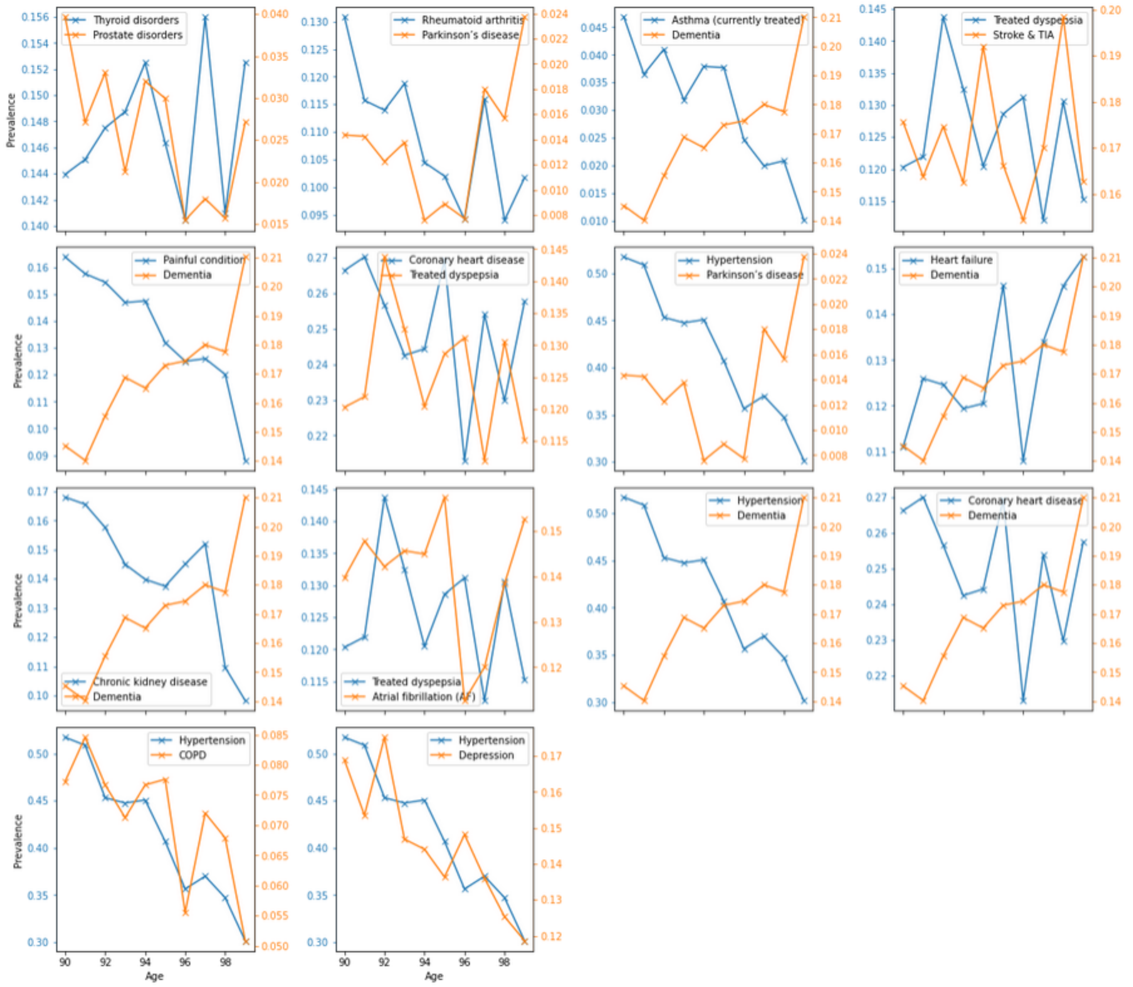

Figure 13: **The effect of the survivor bias on negative associations in the 14 negative associations found in our oldest-old cohort.** Evolution of prevalence with age of pairs of LTCs with negative RR association, where the prevalence is computed with respect to each one-year age group. Some of the negative associations can be a linked to a survivor bias in that the prevalence of some LTCs may shrink as people age because these LTCs increase the likelihood of death and the survivors tend not to have them. An effect of survivor bias on a negative association is hinted by one of the LTCs of the pair declining with age while the other LTC increases with age. This is the case of dementia with asthma, painful condition, chronic kidney disease and hypertension and with Parkinson's disease and hypertension. On the other hand, other LTCs such as dementia do not increase the likelihood of death and is strongly linked to ageing. The combination of these two types of LTCs may be leading to a negative associations. Note that each line has an independent y-axis.

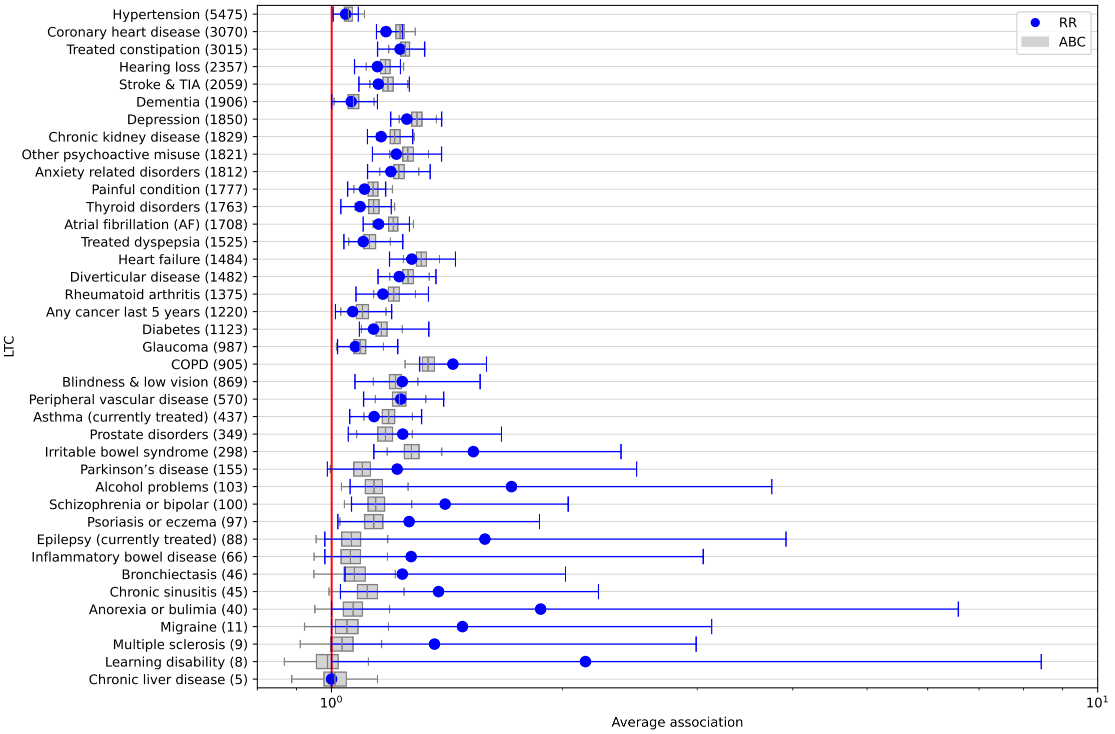

Figure 14: **Average associations by ABC and RR of the 40 LTCs, sorted by their prevalence in the cohort.** The red vertical line corresponds to a score of one, implying that on average there are no associations attached to this LTC. Boxes show the median and interquartile range of the distribution, and whiskers extend to 99% credible intervals. Blue circles denote average associations as computed by RR and error bars extend to the 0.5 and 99.5 percentiles of the estimates from 1000 resamples of the dataset with nonparameteric bootstrap.

References

[1] Jones I, Cocker F, Jose M, Charleston M, Neil AL. Methods of analysing patterns of multimorbidity using network analysis: a scoping review. Journal of Public Health. 2022 jan;(0123456789). Available from: https://doi.org/10.1007/s10389-021-01685-whttps://link.springer.com/10.1007/s10389-021-01685-w.

[2] Fotouhi B, Momeni N, Riolo MA, Buckeridge DL. Statistical methods for constructing disease comorbidity networks from longitudinal inpatient data. Applied Network Science. 2018 dec;3(1):46. Available from: https://appliednetsci.springeropen.com/articles/10.1007/s41109-018-0101-4.

[3] Monchka BA, Leung CK, Nickel NC, Lix LM. The effect of disease co-occurrence measurement on multimorbidity networks: a population-based study. BMC Medical Research Methodology. 2022 jun;22(1):165. Available from: https://doi.org/10.1186/s12874-022-01607-8https://bmcmedresmethodol.biomedcentral.com/articles/10.1186/s12874-022-01607-8.

[4] Kalgotra P, Sharda R, Croff JM. Examining multimorbidity differences across racial groups: a network analysis of electronic medical records. Scientific Reports. 2020 dec;10(1):13538. Available from: https://doi.org/10.1038/s41598-020-70470-8https://www.nature.com/articles/s41598-020-70470-8.

[5] Aguado A, Moratalla-Navarro F, López-Simarro F, Moreno V. MorbiNet: multimorbidity networks in adult general population. Analysis of type 2 diabetes mellitus comorbidity. Scientific Reports. 2020 feb;10(1):2416. Available from: https://www.nature.com/articles/s41598-020-59336-1.

[6] Hernández B, Reilly RB, Kenny RA. Investigation of multimorbidity and prevalent disease combinations in older Irish adults using network analysis and association rules. Sci-

entific Reports. 2019 oct;9(1):14567. Available from: https://www.nature.com/articles/s41598-019-51135-7.

[7] Chmiel A, Klimek P, Thurner S. Spreading of diseases through comorbidity networks across life and gender. New Journal of Physics. 2014 nov;16(11):115013. Available from: https://iopscience.iop.org/article/10.1088/1367-2630/16/11/115013.

[8] Marx P, Antal P, Bolgar B, Bagdy G, Deakin B, Juhasz G. Comorbidities in the diseasome are more apparent than real: What Bayesian filtering reveals about the comorbidities of depression. PLOS Computational Biology. 2017 jun;13(6):e1005487. Available from: https://dx.plos.org/10.1371/journal.pcbi.1005487.

[9] Busija L, Lim K, Szoeke C, Sanders KM, McCabe MP. Do replicable profiles of multimorbidity exist? Systematic review and synthesis. European Journal of Epidemiology. 2019 nov;34(11):1025-53. Available from: https://doi.org/10.1007/s10654-019-00568-5http://link.springer.com/10.1007/s10654-019-00568-5.

[10] Prados-Torres A, Calderón-Larrañaga A, Hancco-Saavedra J, Poblador-Plou B, van den Akker M. Multimorbidity patterns: a systematic review. Journal of Clinical Epidemiology. 2014 mar;67(3):254-66. Available from: https://linkinghub.elsevier.com/retrieve/pii/S0895435613004368.

[11] Kuan V, Denaxas S, Patalay P, Nitsch D, Mathur R, Gonzalez-Izquierdo A, et al. Identifying and visualising multimorbidity and comorbidity patterns in patients in the English National Health Service: a population-based study. The Lancet Digital Health. 2023 jan;5(1):e16-27. Available from: https://linkinghub.elsevier.com/retrieve/pii/S258975002200187X.

[12] Rzhetsky A, Wajngurt D, Park N, Zheng T. Probing genetic overlap among complex human phenotypes. Proceedings of the National Academy of Sciences. 2007 jul;104(28):11694-9. Available from: http://www.pnas.org/cgi/doi/10.1073/pnas.0704820104.

[13] Stan Development Team. 2022. Stan Modeling Language Users Guide and Reference Manual, 2.32.;. Accessed: 2023-06-13. https://mc-stan.org.

[14] Hidalgo CA, Blumm N, Barabási AL, Christakis NA. A Dynamic Network Approach for the Study of Human Phenotypes. PLoS Computational Biology. 2009 apr;5(4):e1000353. Available from: https://dx.plos.org/10.1371/journal.pcbi.1000353.

[15] Clauset A, Newman MEJ, Moore C. Finding community structure in very large networks. Physical Review E. 2004 dec;70(6):066111. Available from: https://link.aps.org/doi/10.1103/PhysRevE.70.066111.
